# Device-measured movement behaviours in over 20,000 China Kadoorie Biobank participants

**DOI:** 10.1186/s12966-023-01537-8

**Published:** 2023-11-24

**Authors:** Yuanyuan Chen, Shing Chan, Derrick Bennett, Xiaofang Chen, Xianping Wu, Yalei Ke, Jun Lv, Dianjianyi Sun, Lang Pan, Pei Pei, Ling Yang, Yiping Chen, Junshi Chen, Zhengming Chen, Liming Li, Huaidong Du, Canqing Yu, Aiden Doherty, on behalf of the China Kadoorie Biobank Collaborative Group

**Affiliations:** 1 Department of Epidemiology and Biostatistics, School of Public Health, Peking University Health Science Center, Beijing, China; 2 Nuffield Department of Population Health, University of Oxford, Oxford, UK; 3 Big Data Institute, Li Ka Shing Centre for Health Information and Discovery, University of Oxford, Oxford, UK; 4 National Institute of Health Research Oxford Biomedical Research Centre, Oxford University Hospitals NHS Foundation Trust, Oxford, UK; 5 Department of Epidemiology and Statistics, Chengdu Medical College, Chengdu, Sichuan China; 6 Sichuan Center for Disease Control and Prevention, Chengdu, Sichuan China; 7 Key Laboratory of Epidemiology of Major Diseases (Peking University), Ministry of Education, Beijing, China; 8 Center for Public Health and Epidemic Preparedness & Response, Peking University, Beijing, China; 9 Medical Research Council Population Health Research Unit, University of Oxford, Oxford, UK; 10 Clinical Trial Service Unit & Epidemiological Studies Unit (CTSU), Nuffield Department of Population Health, University of Oxford, Oxford, UK; 11 China National Center for Food Safety Risk Assessment, Beijing, China

**Keywords:** Movement behaviours, Cohort study, Adults, Accelerometer

## Abstract

**Background:**

Movement behaviours, including physical activity, sedentary behaviour, and sleep have been shown to be associated with several chronic diseases. However, they have not been objectively measured in large-scale prospective cohort studies in low-and middle-income countries. We aim to describe the patterns of device-measured movement behaviours collected in the China Kadoorie Biobank (CKB) study.

**Methods:**

During 2020 and 2021, a random subset of 25,087 surviving CKB individuals participated in the 3^rd^ resurvey of the CKB. Among them, 22,511 (89.7%) agreed to wear an Axivity AX3 wrist-worn triaxial accelerometer for seven consecutive days to assess their habitual movement behaviours. We developed a machine-learning model to infer time spent in four movement behaviours [i.e. sleep, sedentary behaviour, light intensity physical activity (LIPA), and moderate-to-vigorous physical activity (MVPA)]. Descriptive analyses were performed for wear-time compliance and patterns of movement behaviours by different participant characteristics.

**Results:**

Data from 21,897 participants (aged 65.4 ± 9.1 years; 35.4% men) were received for demographic and wear-time analysis, with a median wear-time of 6.9 days (IQR: 6.1–7.0). Among them, 20,370 eligible participants were included in movement behavior analyses. On average, they had 31.1 mg/day (total acceleration) overall activity level, accumulated 7.7 h/day (32.3%) of sleep time, 8.8 h/day (36.6%) sedentary, 5.7 h/day (23.9%) in light physical activity, and 104.4 min/day (7.2%) in moderate-to-vigorous physical activity. There was an inverse relationship between age and overall acceleration with an observed decline of 5.4 mg/day (17.4%) per additional decade. Women showed a higher activity level than men (32.3 vs 28.8 mg/day) and there was a marked geographical disparity in the overall activity level and time allocation.

**Conclusions:**

This is the first large-scale accelerometer data collected among Chinese adults, which provides rich and comprehensive information about device-measured movement behaviour patterns. This resource will enhance our knowledge about the potential relevance of different movement behaviours for chronic disease in Chinese adults.

**Supplementary Information:**

The online version contains supplementary material available at 10.1186/s12966-023-01537-8.

## Introduction

Movement behaviours (moderate-to-vigorous physical activity (MVPA), light-intensity physical activity (LIPA), sedentary behaviour, and sleep) are closely associated with mortality, chronic diseases, cognitive function, and ageing [1–6]. However, in most large-scale population-based studies, movement behaviours are assessed by self-reported questionnaires that are crude and unreliable [7, 8]. Self-reported data suffers from inaccuracy due to recall and reporting bias based on social desirability [9]. Also, LIPA and sedentary behaviours are ubiquitous in our daily lives and are thus unlikely to be recalled precisely [10]. And these limitations may be even more exacerbated among the elderly as they have more difficulty recalling the amount and intensity of activities, especially for LIPA and sedentary behaviours [11]. This lack of precision makes it challenging to recommend the optimal durations and intensities of movement behaviours for health. However, the use of objective techniques (e.g. accelerometers) for assessing movement behaviours offers a chance to overcome some of the limitations of self-report and provides a more accurate reflection of daily patterns of movement behaviours. For example, in the UK Biobank study, the wrist-worn Axivity AX3 accelerometer was used to assess movement behaviours in over 100,000 participants who were asked to wear for 7 days [12].

With the increasing utilization of accelerometers in movement behaviours studies, there remains a significant gap in our understanding of objectively measured movement behaviours among adults in low- and middle-income countries (LMICs). In particular, the limited resources in LMICs often pose a substantial barrier to feasibly conducting comprehensive, large-scale studies using objective techniques. As a consequence, there is a dearth of reliable information about device-measured movement behaviours from East Asian countries, where the evidence is often derived from studies with modest sample sizes and geographically restricted study populations [13–16]. Furthermore, given that genetic profiles, environments, lifestyles, and cultures of East Asian countries are different from the West, movement behaviours may also differ substantially [17, 18]. For example, daytime naps are regarded as part of everyday life for many people in China [19].

The China Kadoorie Biobank (CKB) study is an ongoing large-scale prospective cohort study of over 500,000 participants from five urban and five rural regions. During the 3^rd^ resurvey conducted in 2020–2021 among a random subsample of surviving CKB participants, i.e. ~ 25,000 adults, wrist-worn accelerometers were used to collect device-measured movement behaviours. In this study, we aimed to describe: a) the feasibility of collecting large-scale accelerometer data; b) the free-living movement behaviour patterns and overall activity level; and c) age-, sex- and regional-distribution of different types of movement behaviours in Chinese adults aged 40 and above.

## Methods

### Study population

A general description of the CKB study concerning the study population, baseline data collection, and follow-up procedures has been reported elsewhere [20, 21]. Briefly, trained investigators conducted a baseline survey between 2004–2008. All participants aged 30–79 years that provided written informed consent completed an interviewer-administered questionnaire (collecting information on sociodemographic information, disease history, and major lifestyle factors, including physical activity and sleeping) and a range of physical measurements (e.g., anthropometrics, blood pressure). After the baseline survey, approximately 5% of surviving cohort members from each of the 10 study regions were randomly selected to participate in resurveys every 5–6 years. In the 3^rd^ resurvey conducted between August 2020 and December 2021, in addition to the questionnaire survey and physical measurements, accelerometers were distributed to collect device measurements of movement behaviours [22]. The study protocol of the resurvey was approved by the Peking University Institutional Review Board (No. IRB00001052-18097), and separate written informed consent was obtained from all participants before accelerometer data collection.

### Data collection

The 3^rd^ resurvey of CKB first started in two out of the 10 regions (i.e. Suzhou and Zhejiang) from August to December 2020 and finished in the other eight regions (i.e. Qingdao, Harbin, Haikou, Liuzhou, Sichuan, Gansu, Henan, and Hunan) from March to December 2021. During the survey period, the COVID-19 pandemic was relatively stable and well-managed in our 10 study regions. To minimize the impact, we strategically conducted the field investigation region by region when the local COVID-19 cases were low and the participants lived as usual mostly. In the field work, participants were given an accelerometer (Axivity AX3, Newcastle University, UK) face to face on-site (i.e. before leaving resurvey clinics), and were asked to wear the device on the dominant wrist at all times for seven consecutive days including bathing or swimming (except for extremely high temperature/pressure environments such as in the sauna). The Axivity AX3 is a wrist-worn triaxial accelerometer that has been used in other epidemiological studies such as the UK Biobank [12]. This water-proof device has a dynamic range of ± 8 g and records data at 100 Hz. At the end of the seven days, participants returned the accelerometer to the resurvey coordinating center, where data were downloaded immediately by trained staff using bespoke software developed for the study. In cases where the accelerometer was not returned, the coordinating center staff contacted the participant to confirm its status. After data downloading, the accelerometers were checked, cleaned, and the battery was charged for re-use immediately by trained staff. The process of the accelerometer data collection is shown in supplementary figure 1. Demographic and other variables were obtained through questionnaires and physical examination [22].

### Data processing

We used similar procedures as in the UK Biobank to process raw data and extract information on movement behaviours (https://github.com/activityMonitoring/biobankAccelerometerAnalysis, v6.2.1) [12]. In brief, the processing scheme to extract information from raw accelerometer data included the following steps: (1) resample the acceleration at x/y/z axes to 100 Hz; (2) calibrate the signals of acceleration, and for those with insufficient stationary data recalibrate using data from the previous/next use of the same device; (3) remove sensor noise and gravity; (4) calculate the vector magnitude using the Euclidian Norm minus 1 g (ENMO) with negative values truncated to zero; (5) 30-s epoch analysis; (6) detect non-wear time, i.e. unbroken episodes of at least 60 min during which SD of each axis of acceleration was less than 13.0 mg; (7) calculate the activity volume and intensity summary [12]. To account for potential wear-time diurnal bias, recording interruptions and non-wear-time were imputed using the average values from the corresponding minute of the day on the remaining days of worn data as in the previous study [12].

According to the pre-defined exclusion criteria [12], participants were excluded if (1) the device could not be recalibrated; (2) more than 1% of readings were ‘clipped’ (fell outside the device’s dynamic range of ± 8 g) before or after calibration; (3) there was insufficient wear-time, i.e., participants accumulated less than 3 days (i.e. 72 h) of data or did not have wear-time data in each 1 h of the 24-h cycle; (4) the average acceleration was extremely low (< 1.5 mg) or high (> 100 mg).

### Classification of movement behaviours

Using similar methodology to those used in UK populations, we developed machine-learning classification methods to infer sleep, sedentary behaviour, light intensity activity, and moderate to vigorous intensity physical activity from raw wrist-worn accelerometer data [23]. To inform this development, a validation study was conducted in a Chinese population before the launch of the CKB 3^rd^ resurvey (i.e. in September 2017). This camera-validated dataset (named as CAPTURE-24CN) included 105 adults (non-CKB participants) from Sichuan province. Following a protocol similar to the CAPTURE-24 study in UK adults, [23] participants wore wrist-worn accelerometers and wearable cameras during a 4-day period. Using camera images and time use diaries, trained annotators annotated accelerometer data with labels from the Compendium of Physical Activities [24]. Fine-grained labels were mapped to sleep, sedentary behaviour (e.g., sitting working at a computer, watching television), light physical activity behaviours (e.g., cooking, self-care) and moderate-to-vigorous physical activity behaviours (MVPA; e.g., walking the dog, cycling) [23].

In calculating the activity type and intensity summary, using the CAPTURE-24CN dataset, we developed an accelerometer-based human activity classifier based upon previously validated methods, [23] which used a balanced Random Forest (RF) and a Hidden Markov model (HMM) to infer human activities from acceleration traces obtained from wrist-worn accelerometers with the camera annotations being used as the ground truth. Every 30-s epoch of acceleration trace was first classified into three activities/behaviours: sleep, sedentary behaviours (low-intensity activity in a sitting, lying, or reclining posture), and other non-sedentary behaviours (not meeting any of the other definitions). The non-sedentary behaviours were further sub-classified as either LIPA or MVPA based on acceleration thresholds (ENMO: 100 mg). Further details of the model development and validation are described in the supplementary notes.

### Statistical analysis

Descriptive statistics were generated to report the distribution of demographic characteristics by sex. Frequency and proportions were calculated for categorical variables. Mean and standard deviation were also calculated for age. Age was categorised into 5 groups from 40 years (10-year intervals).

Median and inter-quartile range (IQR) were reported for wear-time compliance overall and in different subgroups. Differences in device wear-time were examined using the Kruskal–Wallis test or Wilcoxon-Mann Whitney test, depending on the number of group variables. To summarise the movement behaviour patterns of different time periods of a 24-h day, we divided time in each day into six-hour quadrants (i.e., 00:00–05:59, 06:00–11:59, etc.)

The amount of movement behaviours was presented in 2 ways: 1) the mean vector magnitude in milli-gravity units (mg) as a measure of overall physical activity, and 2) accumulated time spent in different types of movement behaviours, which constitute a 24-h day in total. Linear regression models were employed to report the distribution of these four movement behaviours by demographic characteristics after adjusting for age, sex, and study region, with the marginal means reported. Considering the positively skewed distribution of MVPA, the logarithmic transformation was utilized in the regression for MVPA. For the linear trend test, the medians of each group were included in the regression model as a continuous variable. For temporal variables (i.e., time of day, day of the week across the 7-day period), the mean and standard error were directly reported without adjustment, and the difference was investigated using one-way repeated-measures ANOVA. For these variables, unadjusted medians with IQR of movement behaviours were calculated. Additionally, we also ran the CKB movement behaviour type classification model on the UKB population to compare the patterns of movement behaviours in both populations.

Statistical analyses were performed using R (version 4.2.1).

## Results

### Study population

**Fig. 1:**
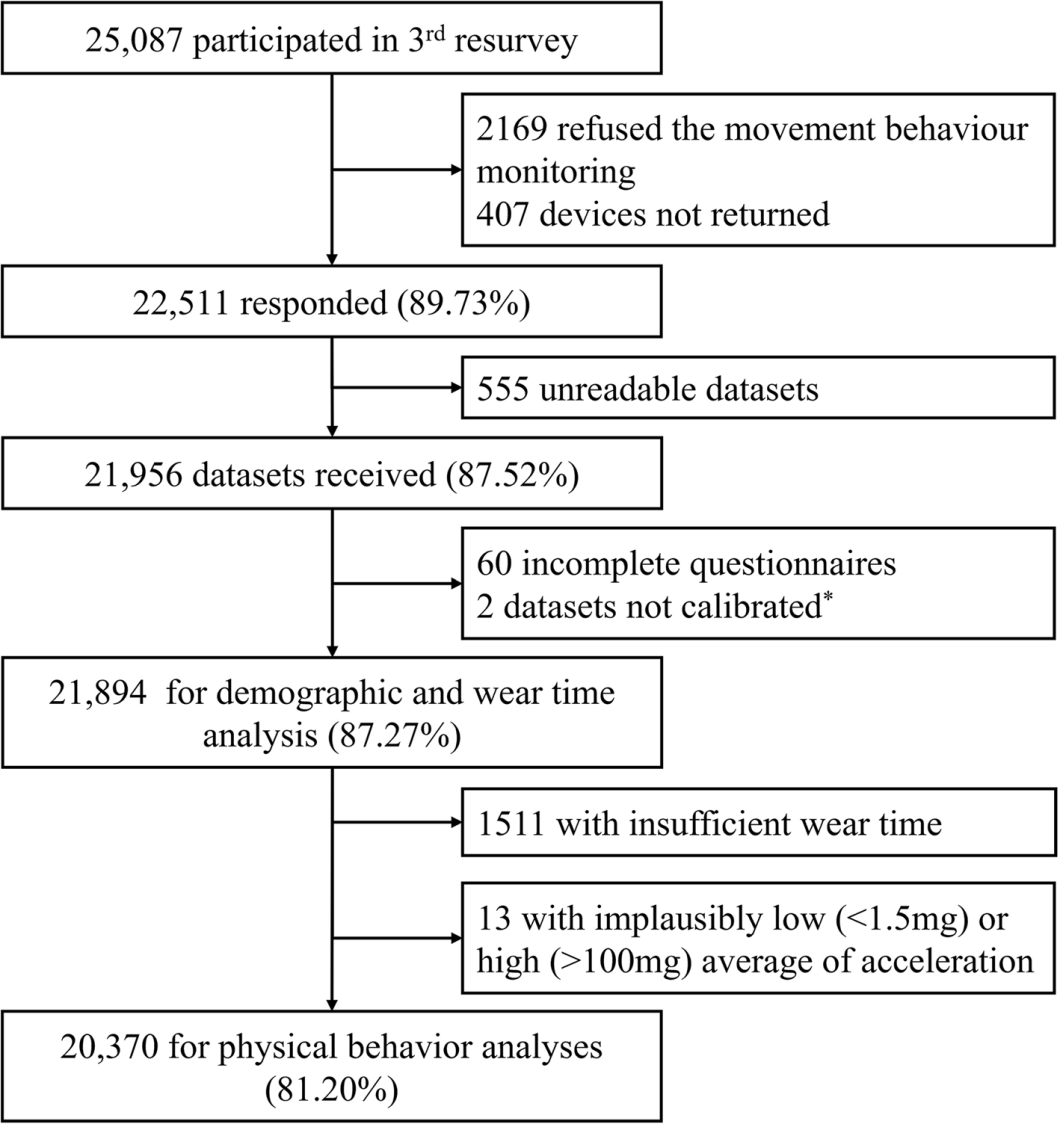
Participant flow chart of the China Kadoorie Biobank accelerometer data collection from 2020–2021. *507 participants with insufficient stationary data, 505 of whom were recalibrated using the previous/next same device by other participants and 2 datasets cannot be calibrated

Between August 2020 and December 2021, 25,087 participants provided written informed consent to participate in the 3^rd^ resurvey of the CKB, of whom 22,918 (91.4%) agreed to wear the accelerometer for seven days and 22,511(89.7%) returned the accelerometer. The cumulative distribution of recruitment into the study is shown in supplementary figure 2. In total, 1850 devices were repeatedly used on a median number of 10 occasions (IQR: 9–17), and 21,956 files were received after excluding 555 unreadable datasets. According to the exclusion criteria mentioned above, two datasets were removed from the analysis as they could not be calibrated using the previous/next same device by different participants. After excluding 60 participants with incomplete questionnaire information, 21,894 remained for demographic and wear-time compliance analysis. Furthermore, 1511 participants had insufficient wear-time, and 13 had implausible acceleration data. Ultimately, 20,370 participants were left in the movement behaviours analysis (Fig. 1).

The characteristics of the study participants by sex are summarised in supplementary table 1. Among them, 35.4% were men, and the mean age was 65.4 ± 9.1 years. Most had been in full-time education for < 12 years (95.4%), and a third were retired (33.2%). Demographic characteristics by participation were shown in supplementary table 2 and were generally similar among responders and non-responders.

### Wear-time analysis

**Fig. 2:**
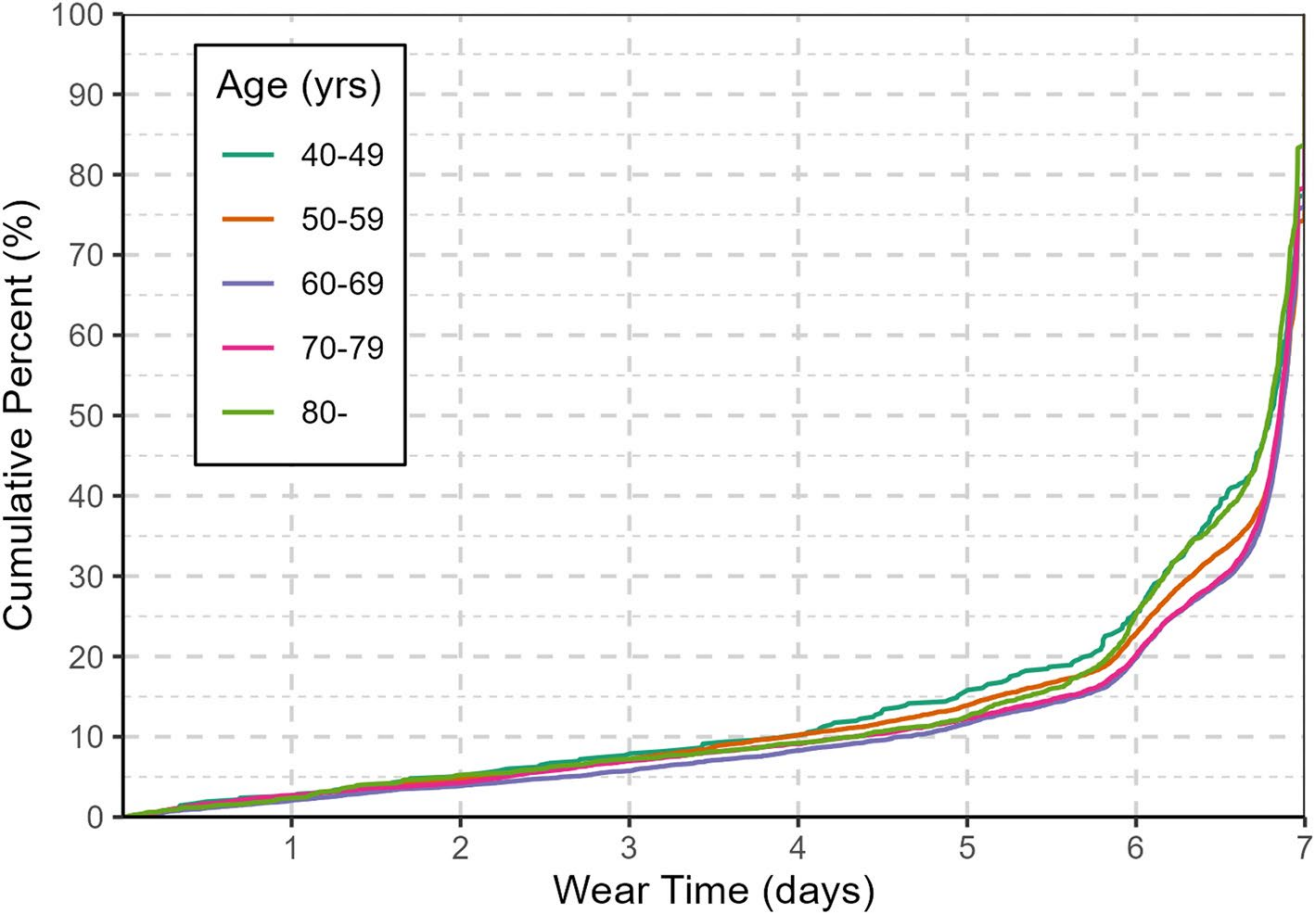
The cumulative distribution function of accelerometer wear-time compliance during the China Kadoorie Biobank accelerometer data collection from 2020–2021. The percent represents what proportion of participants are below the given wear-time criteria. (*n* = 21,894)

Among the total of 21,894 participants eligible for wear-time compliance analysis, the median wear-time was 6.9 days (IQR: 6.1–7.0), and 20,383 (93.1%) participants had sufficient wear-time. In general, the wear-time was similar across basic characteristics, although some regional variations exist, with participants from Haikou wearing the least (i.e. 6.2 days on median and 91.5% had sufficient wear-time). In addition, younger (40–49, 50–59 years) and older (80- years) aged groups had marginally lower levels of compliance (Fig. 2). No gender difference was observed (*P* = 0.712), but married participants had a slightly longer wear-time of 6.9 days (*P* < 0.001) as compared to 6.8 days in non-married participants. People from rural regions generally showed higher participation rates than those from urban regions. Office workers were marginally less compliant than individuals of other occupations, with a median wear-time of 6.7 days (Supplementary table 3). Differences in wear-time distribution were also minimal across time-of-day and day-of-week (Supplementary table 4).

### Movement behaviours analysis

Among the 20,370 participants with valid accelerometer data, the daily overall activity (i.e., overall acceleration) level averaged 31.1 mg, and the average sleep duration accumulated 7.7 h per day. During the recorded waking time, participants spent 8.8 h/d (54%) being sedentary, 35% of waking time engaged in LIPA (5.7 h/d), and 11% (104.4 min/d) in MVPA (Table 1). Medians of these movement behaviours were also calculated and were similar to the mean except for MVPA, due to the positive skewed distribution (Supplementary table 5).

**Table 1:** Distribution of movement behaviours by demographic characteristics [Mean (SE)]

**Variables**^a^	**N (%)**	**Average acceleration (mg/d)**	**MVPA**^**b**^** (min/d)**	**LIPA (h/d)**	**Sedentary (h/d)**	**Sleep (h/d)**
**Age**^**c**^						
40–49	381 (1.9)	36.8 (0.55)	122.0 (0.04)	5.7 (0.10)	8.2 (0.13)	7.6 (0.07)
50–59	6346 (31.2)	35.9 (0.13)	112.9 (0.01)	6.0 (0.02)	8.1 (0.03)	7.6 (0.02)
60–69	7225 (35.5)	31.9 (0.13)	84.4 (0.01)	6.0 (0.02)	8.5 (0.03)	7.7 (0.02)
70–79	5143 (25.2)	26.4 (0.15)	49.5 (0.01)	5.4 (0.03)	9.5 (0.04)	7.9 (0.02)
80-	1275 (6.3)	19.6 (0.30)	20.1 (0.02)	4.4 (0.05)	10.9 (0.07)	8.1 (0.04)
**Sex**						
Men	7079 (34.8)	28.8 (0.13)	67.0 (0.01)	5.0 (0.02)	9.6 (0.03)	7.8 (0.02)
Women	13291 (65.2)	32.3 (0.09)	78.8 (0.01)	6.1 (0.02)	8.3 (0.02)	7.7 (0.01)
**Marital Status**						
Married	16721 (82.1)	31.3 (0.08)	75.8 (0.01)	5.8 (0.02)	8.7 (0.02)	7.7 (0.01)
Others	3649 (17.9)	30.2 (0.18)	68.7 (0.01)	5.5 (0.03)	9.1 (0.04)	7.8 (0.02)
**Education**						
Primary school or below	10371 (50.9)	31.6 (0.12)	75.9 (0.01)	5.9 (0.02)	8.5 (0.03)	7.8 (0.02)
Middle school	9071 (44.5)	30.7 (0.12)	73.2 (0.01)	5.7 (0.02)	9.0 (0.03)	7.7 (0.02)
College or above	928 (4.6)	29.6 (0.38)	71.5 (0.03)	5.2 (0.07)	9.5 (0.09)	7.7 (0.05)
**Household Income Level (yuan/year)**						
< 20,000	3141 (15.4)	30.9 (0.20)	72.3 (0.02)	5.7 (0.04)	8.8 (0.05)	7.8 (0.03)
20,000–75000	9022 (44.3)	31.1 (0.11)	74.1 (0.01)	5.7 (0.02)	8.8 (0.03)	7.7 (0.01)
> 75,000	8207 (40.3)	31.1 (0.13)	75.8 (0.01)	5.8 (0.02)	8.8 (0.03)	7.7 (0.02)
**Occupation**						
Farmers	2542 (12.5)	36.2 (0.24)	108.9 (0.02)	6.3 (0.04)	7.7 (0.06)	7.7 (0.03)
Factory worker	1520 (7.5)	35.8 (0.29)	94.8 (0.02)	6.4 (0.05)	7.6 (0.07)	7.6 (0.04)
Office workers	651 (3.2)	28.6 (0.42)	63.8 (0.03)	5.1 (0.08)	9.7 (0.10)	7.6 (0.06)
Retired	6751 (33.1)	29.2 (0.18)	65.9 (0.01)	5.4 (0.03)	9.3 (0.04)	7.7 (0.02)
House wife/husband	6198 (30.4)	30.2 (0.16)	72.6 (0.01)	5.7 (0.03)	8.8 (0.04)	7.9 (0.02)
Service workers/Self-employed	1695 (8.3)	32.8 (0.27)	82.9 (0.02)	6.1 (0.05)	8.3 (0.06)	7.7 (0.04)
Unemployed & others	1013 (5.0)	27.9 (0.34)	48.6 (0.03)	5.0 (0.06)	9.7 (0.08)	7.8 (0.04)
**BMI (kg/m**^**2**^**)**^**c**^						
< 18.5	623 (3.1)	31.6 (0.42)	71.5 (0.03)	5.6 (0.08)	8.6 (0.10)	7.9 (0.06)
18.5–23.9	8589 (42.2)	32.3 (0.11)	81.1 (0.01)	5.8 (0.02)	8.4 (0.03)	7.8 (0.02)
24–27.9	8042 (39.5)	30.8 (0.12)	74.0 (0.01)	5.7 (0.02)	8.8 (0.03)	7.7 (0.02)
≥ 28	3106 (15.3)	28.5 (0.19)	60.4 (0.01)	5.4 (0.04)	9.6 (0.05)	7.5 (0.03)

*MVPA* Moderate-to-vigorous physical activity, *LIPA* Light physical activity, *mg/d* milli-gravity units per day, *h/d* hours per day

^a^Values were adjusted for age, sex, and region (where appropriate)

^b^Level of MVPA was reported based on logarithmic transformation

^c^For all movement behaviours, *P*_Linear trend_ < 0.001

**Fig. 3:**
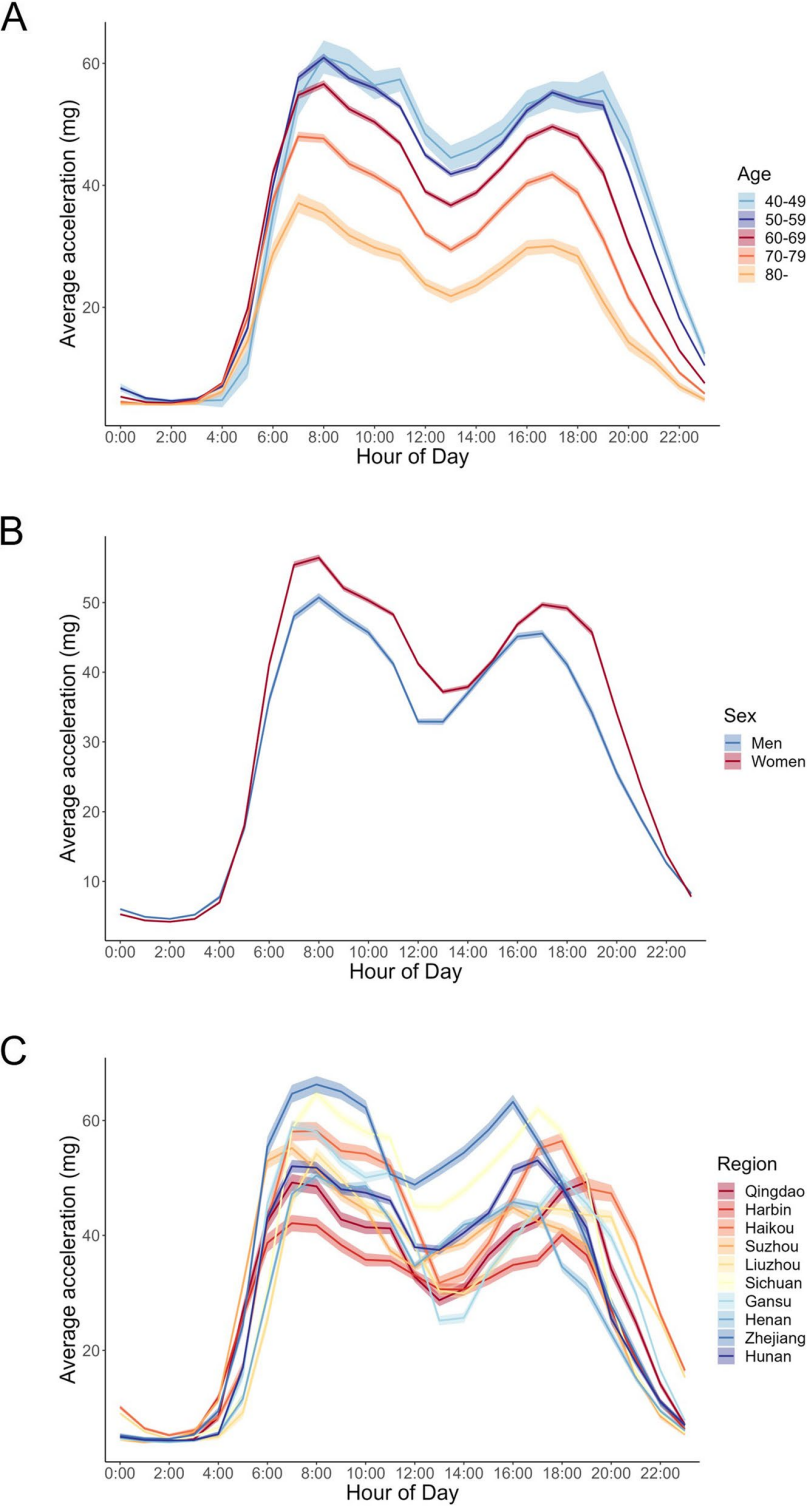
24-h profile of average acceleration in 20,370 China Kadoorie Biobank participants in 2020–2021 by **A**) sex; **B**) age group, and **C**) region. Values were adjusted for age, sex and region (where appropriate). Ribbon represents the 95% confidence interval

Overall activity levels were generally highest in the younger group and progressively lower with age (Table 1 and Fig. 3A), with an average of 5.4 mg/day (17.4%) lower activity by decade of aging. In particular, time spent on MVPA decreased 23.4 min/day (9.0%), LIPA decreased 29.2 min/day (8.5%) but sedentary time increased 55.8 min/day (10.6%) on average between individuals for every ten years of age increase (for all movement behaviours, *P*_Linear trend_ < 0.001, Supplementary figure 3). Participants aged 80 years or older had the longest (8.1 h/d) sleep time. For every decade older of age, total sleep time was 7.0 min/day (1.5%) higher (Table 1).

Women showed a higher activity level than men (Fig. 3B), with a longer time of physical activity (65.6 min/d longer for LIPA, 19.3 min/d longer for MVPA), and shorter time of sedentary behaviour (79.8 min/d shorter) (Table 1 and Supplementary figure 4, all *P* < 0.001). Married people tended to have a higher level of overall activity than those living without a partner (*P* < 0.001, Table 1), and this is the case in both men and women (data not shown).

**Fig. 4:**
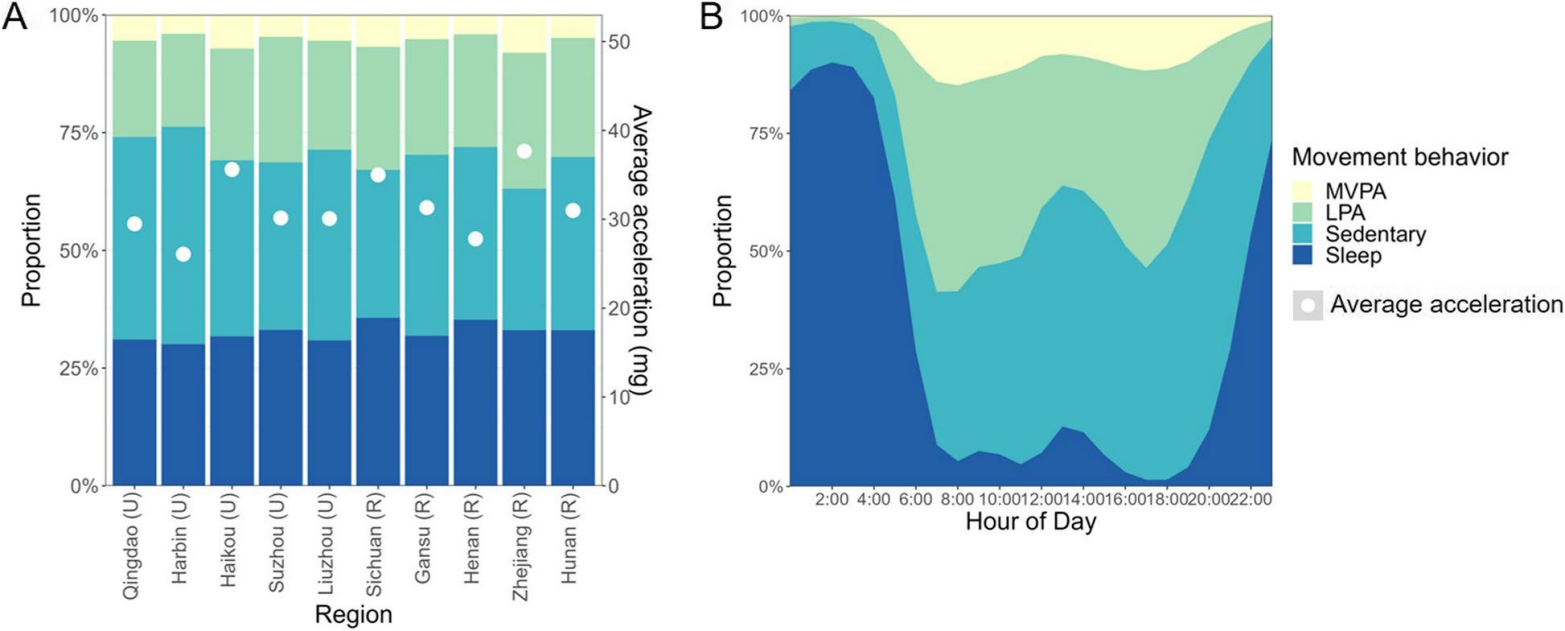
Time composition of different movement behaviour types in 20,370 China Kadoorie Biobank participants in 2020–2021 by **A**) different regions and **B**) hour-of-day. Time spent in four types of movement behaviour, which constitute a 24-h day in total, are represented by colour shading. The white point (4A) represents the level of average acceleration (mg). Values by region were adjusted for age and sex. The level of MVPA was reported based on logarithmic transformation

Results indicated a marked disparity in the overall activity level and time allocation of movement behaviours among people across different regions (Figs. 3C and 4A). There was heterogeneity in the proportion of participants who engaged in daytime naps and the duration of these naps across the ten regions (Supplementary figure 5). People from urban regions had a lower overall activity, MVPA, LIPA, and sleep duration. However, they spent much more time on sedentary behaviour compared to those from rural regions (Supplementary table 6, all *P* < 0.001). Movement behaviour patterns differed significantly among different occupations, where rural farmers and factory workers had the highest level of MVPA (108.9 min/d and 94.8 min/d, respectively on average), much more than office workers. In addition, people with normal BMI were on average more likely to have higher overall activity levels and were more frequently involved in physical activity (both MVPA and LIPA). A decrease of 3.7 mg/day (11.8%) in average acceleration and an increase of 74.1 min/d (14.1%) in sedentary time were also observed by 10 units higher BMI (*P*_Linear trend_ < 0.001 for all movement behaviours).

Time spent in MVPA and sleep was slightly longer on weekdays than during weekends (Supplementary table 7, *P* ≤ 0.002). As shown in the Fig. 3, the most active period of day was around 6:00–10:00 and 16:00–18:00, with an apparent lower activity level at noon (12:00–14:00), in accordance with a higher proportion of napping (Fig. 4B). Daily activity among participants tended to initiate between 5:00 and 7:00. Two peaks for MVPA were around 8:00 and 18:00. There were three peaks for LIPA with 2 major peaks at 7:00 and 17:00 as well as a minor peak at 11:00 (Supplementary figure 3). The time pattern of overall activity level was similar across all age groups (Fig. 3A) and sex (Fig. 3B), with a minor lag in women and the youngest age group (40–49 yrs) in the evening.

Regarding the comparison between our study (CKB) and the UK Biobank (UKB), participants in UKB were approximately three years younger than those in CKB and the sex distribution was more balanced (Supplementary table 8). Using our validated model, the UKB population had an overall activity level of 27.1 mg, accumulated 7.8 h/d of sleep time, 10.2 h/d sedentary, 4.3 h/d in LIPA, and 88.1 min/d in MVPA (Supplementary table 9). Comparing supplementary tables 5 and 9, younger CKB participants appeared more active than younger UKB participants. For example, comparing those aged 40 to 49, CKB participants appeared to have higher median acceleration (35.5 vs 30.5 mg), LIPA (5.7 vs 4.1 h/d) and MVPA (129 vs 106 min/d). However older UKB participants appeared to have higher median MVPA time than older CKB participants. When considering those aged 70 to 79, acceleration medians appeared similar (24.9 vs 24.2 mg), UKB participants were more sedentary (10.5 vs 9.5 h/day), CKB participants spent more time in LIPA (5.3 vs 4.4 h/day), and UKB participants were observed to have slightly more MVPA time (69 vs 59 min/d).

## Discussion

In this large-scale epidemiological study of device-measured movement behaviours in Chinese adults, we found very high response (89.7%) and adherence (93.1%) rates with most participants having sufficient wear-time to support epidemiological analysis. Using separate free-living ‘ground truth’ data, we developed machine-learning methods to reliably classify movement behaviours from wrist-worn accelerometer data in Chinese adults. By applying these methods, we were able to accurately describe movement behaviours in 20,370 China Kadoorie Biobank participants.

Generally, participants in this study showed high compliance with the instruction in participation and wear-time. The response rate for the CKB was notably higher than that of the UKB, with rates of 89.7% and 44.2% [12], respectively. This discrepancy may be partially attributed to the fact that CKB participants were provided with their devices in person, rather than via mail in UKB during the deployment phase. We found that demographic characteristics were similar between responders and non-responders, suggesting that selection bias is likely to be negligible.

Compared with results from previous study on UKB used the same device [23], we found longer MVPA and LIPA, but shorter sleep and sedentary time in the CKB. When we updated the UKB results with our CKB model, the results in MVPA and sleep time were similar, but differences in LIPA and sedentary time were more prominent (5.7 vs 4.3 h/d for LIPA, 8.7 vs 10.2 h/d for sedentary). And a more pronounced age trend was observed as well in CKB participants where younger people spent more time in MVPA and older people spent slightly less time in MVPA than equivalently aged participants in the UK Biobank (Supplementary table 5 and 9). These differences could be partly attributed to the different socio-demographic characteristic, such as age and occupation of the study sample. More than a half of the CKB participants were rural residents (58.6%) and 20% of participants were still engaged in manufactory work (farmers or factory workers). Moreover, our results showed that in CKB there was a “napping window” during 12:00–15:00, where levels of activity were apparently lower and the proportions of sleep were high. These results were in agreement with the information extracted from the questionnaire in our previous work that some people in China are used to taking extra daytime napping at noon [19].

The decreases in overall acceleration, moderate, and light activity, as well as the increase in sedentary and total sleep time by older age group in our study were similar to those previously reported in studies from both eastern [25] and western countries [26]. The sex-difference in overall activity level observed in our study (i.e. higher in women than in men) was in contrast to the subjective findings from the questionnaire-based studies in Chinese [27] and other populations [28]. However, it was in agreement with results in the UK Biobank and some other studies using the accelerometer to assess movement behaviours in Western [29] and Asian countries [30]. This difference was mainly driven by light activity, including a large proportion of household activities which are usually undertaken by women and are hard to be recalled [10]. Detailed comparisons are still needed to better understand the extent of mis-reporting on physical activity amounts in Chinese men and women [31]. Besides, our results showed that there was a great variety in movement behaviours across 10 study regions. This variability is likely due to a range of factors, including seasonal differences at measuring, as well as geographical variations in weather patterns, sunshine duration, temperature, and lifestyle factors. As such, later investigations should consider these geographical variation and seasonal difference when associating accelerometer data with incident disease in the future.

The strengths of this study include a large sample size, high response rate, great wear-time compliance and population-specific validation process which ensured the reliability and validity of this data. However, some limitations need to be accounted for. First, the COVID-19 pandemic posed a challenge for our field work during the 3^rd^ resurvey. To minimize the impact of the pandemic on the activity of the participants, we had to adjust the investigation periods differently according to the local situation of each region. This resulted in different seasons for the fieldwork in different regions, which may have confounded the geographical variations with the seasonal variations. Second, a 7-day measurement period may not represent longer-term habitual movement behaviours throughout the year. However, a previous study has shown relatively low within-person variation and good reproducibility in Western populations, where intraclass correlation coefficients ranged from 0.67 for bouted-MVPA to 0.82 for total daily counts [32]. Third, objectively measured movement behaviours in this study were based on wrist-worn accelerometers, which may have limitations on detecting certain postures or efforts that require greater energy expenditure, such as carrying loads. Fourth, the study cohort was not designed to be nationally representative. However, within each study region, the participants were relatively unselected.

## Conclusions

In conclusion, we have described device-measured movement behaviours in a prospective Chinese cohort study. We found very high response and adherence rates and subsequent data analysis showed good face validity. As follow-up data becomes available, the China Kadoorie Biobank is well placed to contribute to improvements in our understanding of how movement behaviours are associated with health outcomes in older Chinese adults. We hope that this study will inspire cohort studies in other LMICs to collect device-measured movement behaviours at scale.

### Supplementary Information

**Additional file 1:** Supplementary: Members of the China Kadoorie Biobank collaborative group. **Supplementary Note.** Model development and validation. **Supplementary Table 1.** Characteristics of the China Kadoorie Biobank accelerometer data collection from 2020-2021 [N(%)]. **Supplementary Table 2.** Demographics of those who participated versus those who did not [N(%)]. **Supplementary Table 3.** Wear-time compliance of the study population by demographic characteristics (*N*=21,894). The maximum possible wear time is 7.0 days. **Supplementary Table 4.** Wear-time compliance of the study population by temporal characteristics (*N*=21,894). **Supplementary Table 5.** Median (IQR) levels of movement behaviours (*N*=20,370). **Supplementary Table 6.** Mean (SE) levels of movement behaviours by regions ^a^ (*N*=20,370). **Supplementary Table 7.** Mean (SE) levels of movement behaviours by temporal characteristics (*N*=20,370). **Supplementary Table 8.** Characteristics of the UK Biobank accelerometer dataset [N(%)]. **Supplementary Table 9.** Median (IQR) levels of movement behaviours in the UK Biobank accelerometer dataset (*N*=96,313). **Supplementary Table 10.** Confusion matrices of the machine learning classifier in free-living environments: the CAPTURE-24CN and CAPTURE-24 studies. Minutes shown in brackets. **Supplementary Figure 1.** Flowchart of the process of the accelerometer data collection. **Supplementary Figure 2.** Start/end date of fieldwork across 10 study regions*. **Supplementary Figure 3.** 24-h profile of four movement behaviours by age group^*^. **Supplementary Figure 4.** 24-h profile of four movement behaviours by sex. **Supplementary Figure 5.** 24-h profile of different movement behaviours by region^*^. **Supplementary Figure 6.** The process of model development, validation and deployment.

## Data Availability

Data are available upon reasonable request. Details of how to access China Kadoorie Biobank data and details of the data release schedule are available from www.ckbiobank.org/site/Data+Access.

## Abbreviations

BMI: Body mass index
CKB: China Kadoorie Biobank study
LIPA: Light intensity physical activity
LMICs: Low- and middle-income countries
MVPA: Moderate-to-vigorous physical activity
UKB: UK Biobank study
