# Correction: Device-measured movement behaviours in over 20,000 China Kadoorie Biobank participants

**DOI:** 10.1186/s12966-024-01597-4

**Published:** 2024-05-01

**Authors:** Yuanyuan Chen, Shing Chan, Derrick Bennett, Xiaofang Chen, Xianping Wu, Yalei Ke, Jun Lv, Dianjianyi Sun, Lang Pan, Pei Pei, Ling Yang, Yiping Chen, Junshi Chen, Zhengming Chen, Liming Li, Huaidong Du, Canqing Yu, Aiden Doherty, the China Kadoorie Biobank Collaborative Group

**Affiliations:** 1 Department of Epidemiology and Biostatistics, School of Public Health, Peking University Health Science Center, Beijing, China; 2 Nufeld Department of Population Health, University of Oxford, Oxford, UK; 3 Big Data Institute, Li Ka Shing Centre for Health Information and Discovery, University of Oxford, Oxford, UK; 4 National Institute of Health Research Oxford Biomedical Research Centre, Oxford University Hospitals NHS Foundation Trust, Oxford, UK; 5 Department of Epidemiology and Statistics, Chengdu Medical College, Chengdu, Sichuan China; 6 Sichuan Center for Disease Control and Prevention, Chengdu, Sichuan China; 7 Key Laboratory of Epidemiology of Major Diseases (Peking University), Ministry of Education, Beijing, China; 8 Center for Public Health and Epidemic Preparedness & Response, Peking University, Beijing, China; 9 Medical Research Council Population Health Research Unit, University of Oxford, Oxford, UK; 10 Clinical Trial Service Unit & Epidemiological Studies Unit (CTSU), Nufeld Department of Population Health, University of Oxford, Oxford, UK; 11 China National Center for Food Safety Risk Assessment, Beijing, China

**Correction:** **Int J Behav Nutr Phys Act 20, 138 (2023)**

**https://doi.org/10.1186/s12966-023-01537-8**

In the Original Article [1], the values for the BMI groups " < 18.5" and "18.5-23.9" were mistakenly swapped. The correct values should be as follows:


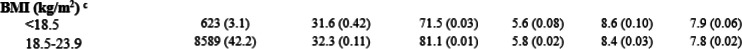


Moreover, the 4th paragraph of the ‘Movement behaviours analysis’ section in ‘Results’ mentioned incorrect statement as follows: "In addition, people with a lower BMI were on average more likely to have higher overall activity levels and were more frequently involved in physical activity (both MVPA and LIPA)."

The correct sentence should have been: "In addition, people with a normal BMI were on average more likely to have higher overall activity levels and were more frequently involved in physical activity (both MVPA and LIPA)."

Lastly, in the Supplementary, the first sentence of the “Evaluation of the model” was “The refined model achieved a Cohen’s kappa score of 0.667 (0.605, 0.749) and an accuracy of 0.811 (0.787, 0.853) on the CAPTURE-24CN dataset”.

The correct statement should have been: "The refined model achieved a Cohen’s kappa score of 0.674 (0.614, 0.743) and an accuracy of 0.814 (0.783, 0.851) on the CAPTURE-24CN dataset. "

The original article has been updated.
