# Supplementary material for: Device-measured movement behaviours in over 20,000 China Kadoorie Biobank participants
Source: Int J Behav Nutr Phys Act. 2023 Nov 24;20:138. doi: 10.1186/s12966-023-01537-8 (PMC10668372; doi:10.1186/s12966-023-01537-8)
Supplement: Supplementary file 1 — Additional file 1: Supplementary: Members of the China Kadoorie Biobank collaborative group. Supplementary Note. Model development and validation. Supplementary Table 1. Characteristics of the China Kadoorie Biobank accelerometer data collection from 2020-2021 [N(%)]. Supplementary Table 2. Demographics of those who participated versus those who did not [N(%)]. Supplementary Table 3. Wear-time compliance of the study population by demographic characteristics (N=21,894). The maximum possible wear time is 7.0 days. Supplementary Table 4. Wear-time compliance of the study population by temporal characteristics (N=21,894). Supplementary Table 5. Median (IQR) levels of movement behaviours (N=20,370). Supplementary Table 6. Mean (SE) levels of movement behaviours by regions a (N=20,370). Supplementary Table 7. Mean (SE) levels of movement behaviours by temporal characteristics (N=20,370). Supplementary Table 8. Characteristics of the UK Biobank accelerometer dataset [N(%)]. Supplementary Table 9. Median (IQR) levels of movement behaviours in the UK Biobank accelerometer dataset (N=96,313). Supplementary Table 10. Confusion matrices of the machine learning classifier in free-living environments: the CAPTURE-24CN and CAPTURE-24 studies. Minutes shown in brackets. Supplementary Figure 1. Flowchart of the process of the accelerometer data collection. Supplementary Figure 2. Start/end date of fieldwork across 10 study regions*. Supplementary Figure 3. 24-h profile of four movement behaviours by age group*. Supplementary Figure 4. 24-h profile of four movement behaviours by sex. Supplementary Figure 5. 24-h profile of different movement behaviours by region*. Supplementary Figure 6. The process of model development, validation and deployment. [file 12966_2023_1537_MOESM1_ESM.docx]

# Supplementary

## Members of the China Kadoorie Biobank collaborative group:

**International Steering Committee:** Junshi Chen, Zhengming Chen (PI), Robert Clarke, Rory Collins, Yu Guo, Liming Li (PI), Jun Lv, Richard Peto, Robin Walters. **International Co-ordinating Centre, Oxford:** Daniel Avery, Derrick Bennett, Ruth Boxall, Sue Burgess, Ka Hung Chan, Yumei Chang, Yiping Chen, Zhengming Chen, Johnathan Clarke; Robert Clarke, Huaidong Du, Ahmed Edris Mohamed, Zammy Fairhurst-Hunter, Hannah Fry, Mike Hill, Michael Holmes, Pek Kei Im, Andri Iona, Maria Kakkoura, Christiana Kartsonaki, Rene Kerosi, Kuang Lin, Mohsen Mazidi, Iona Millwood, Sam Morris, Qunhua Nie, Alfred Pozarickij, Paul Ryder, Saredo Said, Dan Schmidt, Paul Sherliker, Becky Stevens, Iain Turnbull, Robin Walters, Lin Wang, Neil Wright, Ling Yang, Xiaoming Yang, Pang Yao. **National Co-ordinating Centre, Beijing:** Yu Guo, Xiao Han, Can Hou, Jun Lv, Pei Pei, Chao Liu, Canqing Yu, Qingmei Xia.

**10 Regional Co-ordinating Centres: Qingdao CDC:** Zengchang Pang, Ruqin Gao, Shanpeng Li, Haiping Duan, Shaojie Wang, Yongmei Liu, Ranran Du, Yajing Zang, Liang Cheng, Xiaocao Tian, Hua Zhang, Yaoming Zhai, Feng Ning, Xiaohui Sun, Feifei Li. **Licang CDC:** Silu Lv, Junzheng Wang, Wei Hou. **Heilongjiang Provincial CDC:** Wei Sun, Shichun Yan, Xiaoming Cui. **Nangang CDC:** Chi Wang, Zhenyuan Wu,Yanjie Li, Quan Kang. **Hainan Provincial CDC:** Huiming Luo, Tingting Ou. **Meilan CDC:** Xiangyang Zheng, Zhendong Guo, Shukuan Wu, Yilei Li, Huimei Li. **Jiangsu Provincial CDC:** Ming Wu, Yonglin Zhou, Jinyi Zhou, Ran Tao, Jie Yang, Jian Su. **Suzhou CDC:** Fang Liu, Jun Zhang, Yihe Hu, Yan Lu, Liangcai Ma, Aiyu Tang, Shuo Zhang, Jianrong Jin, Jingchao Liu. **Guangxi Provincial CDC:** Mei Lin, Zhenzhen Lu. **Liuzhou CDC:** Lifang Zhou, Changping Xie, Jian Lan,Tingping Zhu,Yun Liu, Liuping Wei, Liyuan Zhou, Ningyu Chen, Yulu Qin, Sisi Wang. **Sichuan Provincial CDC:** Xianping Wu, Ningmei Zhang, Xiaofang Chen, Xiaoyu Chang. **Pengzhou CDC:** Mingqiang Yuan, Xia Wu, Xiaofang Chen, Wei Jiang, Jiaqiu Liu, Qiang Sun. **Gansu Provincial CDC:** Faqing Chen, Xiaolan Ren, Caixia Dong. **Maiji CDC:** Hui Zhang, Enke Mao, Xiaoping Wang, Tao Wang, Xi zhang. **Henan Provincial CDC:** Kai Kang, Shixian Feng, Huizi Tian, Lei Fan. **Huixian CDC:** XiaoLin Li, Huarong Sun, Pan He, Xukui Zhang. **Zhejiang Provincial CDC:** Min Yu, Ruying Hu, Hao Wang. **Tongxiang CDC**: Xiaoyi Zhang, Yuan Cao, Kaixu Xie, Lingli Chen, Dun Shen. **Hunan Provincial CDC:** Xiaojun Li, Donghui Jin, Li Yin, Huilin Liu, Zhongxi Fu. **Liuyang CDC:** Xin Xu, Hao Zhang, Jianwei Chen,Yuan Peng, Libo Zhang, Chan Qu.

## Supplementary Note: Model development and validation.

### Participants of CAPTURE-24CN dataset

CAPTURE-24, an accelerometer and wearable camera dataset of 152 adults aged 18–91 in 2014–2015 was used to develop and validate accelerometer-only machine-learning classification methods for use in Western populations^1^. Similarly, in 2017, CAPTURE-24CN, an accelerometer and wearable camera dataset of 105 adults aged 20-84 (mean[SD]: 47.4[12.1] years) was also collected in Sichuan province of China. In order to tailor the model to a Chinese population, the CAPTURE-24CN and CAPTURE-24 datasets were both employed. 61 participants of CAPTURE-24CN were excluded if their accelerometer could not be calibrated or had recording errors, delivered less than 24 hrs of total wear-time, and/or had less than 4 hrs of camera-annotated time. This resulted in 44 remaining participants for model validation/testing. As we did not have sleep information during accelerometer wear periods, we pragmatically assigned sleep intervals between 00:00-06:00 following a visual inspection of the acceleration trace.

### The machine-learning method of UK Biobank study

In the activity classification process, balanced random forests and hidden Markov models (HMMs) were employed. The balanced random forests were introduced as an alternative to the traditional random forest algorithm to address the issue of imbalanced samples and enhance classification performance, particularly for the minority class (e.g., MVPA in this study). However, balanced random forests solely consider the features of individual data points, disregarding their sequential relationship. Consequently, a smoothing technique, such as the hidden Markov model, was required to prevent implausible sequences generated by the balanced random forests (e.g., brief MVPA periods interspersed with long sleep durations). Within the HMM framework, the predicted labels from balanced random forests were treated as observable emissions of concealed true states, adhering to the Markov chain property. More details of these methods could be referred to in the previous work^2^.

### Retraining of the classifier

Based on the machine-learning method of the UK Biobank, we retrained the classifier to identify behaviours more robustly in both datasets. For example, walking and bicycling activities in the CAPTURE-24 dataset accounted for 82% of all MVPA instances, as almost all participants in this dataset were office workers. In contrast, participants in the CAPTURE-24CN dataset consisted mainly of rural and/or manual workers, contributing more varied MVPA instances -- only 42% of MVPA instances were walking or bicycling activities, and the rest were occupational MVPA (e.g. farm work, construction work) that were harder to classify using the original model.

In light of this, we reworked the classifier and activity label set as follows (Supplementary Figure 6): A balanced random forest and a Hidden Markov model were used to first classify activities as either "sleep", "sedentary, or "other non-sedentary". Then, "other non-sedentary" instances are further subclassified as either “light-intensity physical activity” (LIPA) or “moderate-to-vigorous physical activity” (MVPA) using simple acceleration cut-points used in the literature: If ENMO ≥ 100 mg (truncated Euclidean norm minus one) then reassign as MVPA, else reassign as LIPA.

### Evaluation of the model

The refined model achieved a Cohen’s kappa score of 0.674 (0.614, 0.743) and an accuracy of 0.814 (0.783, 0.851) on the CAPTURE-24CN dataset. On the CAPTURE-24 dataset, the model achieves a Cohen’s kappa score of 0.840 (0.823, 0.855) and an accuracy CAPTURE-24CN of 0.900 (0.891, 0.913), estimated using leave-one-out cross-validation. The respective confusion matrices are shown in Supplementary Table 10. We observe that differences in the model performance across both datasets were most noticeable at the boundary between “sedentary” and “other non-sedentary”. Visual inspection of the camera images revealed that it was indeed difficult for a human annotator to determine whether non-sedentary activities took place (e.g. walking vs standing still) due to the poor camera frame rate.

### Reference

1 Doherty A, Smith-Byrne K, Ferreira T, *et al.* GWAS identifies 14 loci for device-measured physical activity and sleep duration. *Nat Commun* 2018;**9**. doi:10.1038/s41467-018-07743-4

2 Walmsley R, Chan S, Smith-Byrne K, *et al.* Reallocation of time between device-measured movement behaviours and risk of incident cardiovascular disease. *Br J Sports Med* 2021;:bjsports-2021-104050. doi:10.1136/bjsports-2021-104050

## Supplementary Table 1. Characteristics of the China Kadoorie Biobank accelerometer data collection from 2020-2021 [N(%)].

|  |  | **Men** | **Women** | **Overall** |
| --- | --- | --- | --- | --- |
| **N (%)** | | 7756 (35.4) | 14,138 (64.6) | 21,894 |
| **Age (yrs)** | |  |  |  |
|  | Mean (SD) | 66.0 (9.2) | 65.0 (9.0) | 65.4 (9.1) |
|  | 40-49 | 124 (1.6) | 293 (2.1) | 417 (1.9) |
|  | 50-59 | 2242 (28.9) | 4624 (32.7) | 6866 (31.4) |
|  | 60-69 | 2709 (34.9) | 4983 (35.2) | 7692 (35.1) |
|  | 70-79 | 2113 (27.2) | 3428 (24.2) | 5541 (25.3) |
|  | 80- | 568 (7.3) | 810 (5.7) | 1378 (6.3) |
| **Region** | |  |  |  |
|  | Qingdao | 400 (5.2) | 1173 (8.3) | 1573 (7.2) |
|  | Harbin | 584 (7.5) | 1066 (7.5) | 1650 (7.5) |
|  | Haikou | 378 (4.9) | 881 (6.2) | 1259 (5.8) |
|  | Suzhou | 938 (12.1) | 1294 (9.2) | 2232 (10.2) |
|  | Liuzhou | 758 (9.8) | 1574 (11.1) | 2332 (10.7) |
|  | Sichuan | 957 (12.3) | 1841 (13.0) | 2798 (12.8) |
|  | Gansu | 874 (11.3) | 1676 (11.9) | 2550 (11.6) |
|  | Henan | 1254 (16.2) | 1964 (13.9) | 3218 (14.7) |
|  | Zhejiang | 506 (6.5) | 954 (6.7) | 1460 (6.7) |
|  | Hunan | 1107 (14.3) | 1715 (12.1) | 2822 (12.9) |
| **Married** | | 6952 (89.6) | 400 (5.2) | 17977 (82.1) |
| **Education** | |  |  |  |
|  | Primary school or below | 3250 (41.9) | 7845 (55.5) | 11095 (50.7) |
|  | Middle school | 4030 (52.0) | 5763 (40.8) | 9793 (44.7) |
|  | College or above | 476 (6.1) | 530 (3.7) | 1006 (4.6) |
| **Household Income Level (yuan/year)** | | |  |  |
|  | <20000 | 1174 (15.1) | 2194 (15.5) | 3368 (15.4) |
|  | 20000-75000 | 3279 (42.3) | 6394 (45.2) | 9673 (44.2) |
|  | >75000 | 3303 (42.6) | 5550 (39.3) | 8853 (40.4) |
| **Occupation** | |  |  |  |
|  | Farmers | 1167 (15.0) | 1549 (11.0) | 2716 (12.4) |
|  | Factory workers | 1094 (14.1) | 574 (4.1) | 1668 (7.6) |
|  | Office workers | 479 (6.2) | 240 (1.7) | 719 (3.3) |
|  | Retired | 2408 (31.0) | 4869 (34.4) | 7277 (33.2) |
|  | House wife/husband | 1032 (13.3) | 5547 (39.2) | 6579 (30.0) |
|  | Service workers/Self-employed | 776 (10.0) | 1047 (7.4) | 1823 (8.3) |
|  | Unemployed & others | 800 (10.3) | 312 (2.2) | 1112 (5.1) |
| **BMI (kg/m^2^)^*^** | |  |  |  |
|  | <18.5 | 249 (3.2) | 429 (3.0) | 678 (3.1) |
|  | 18.5-24 | 3394 (43.8) | 5891 (41.7) | 9285 (42.4) |
|  | 24-28 | 3077 (39.7) | 5534 (39.2) | 8611 (39.3) |
|  | >28 | 1032 (13.3) | 2278 (16.1) | 3310 (15.1) |

^*^10 participants with missing value for BMI.

## Supplementary Table 2. Demographics of those who participated versus those who did not [N(%)].

| **Characteristics** | | **Yes ^a^** | **No** | | **Overall** |
| --- | --- | --- | --- | --- | --- |
|  |  |  | **declined** | **not returned the accelerometer** |  |
| **N (%)** | | 22,511 (89.7) | 2169 (8.6) | 407 (1.6) | 25,087 |
| **Sex** |  |  |  |  |  |
|  | Men | 7951 (88.5) | 876 (9.8) | 156 (1.7) | 8983 (35.8) |
|  | Women | 14,560 (90.4) | 1293 (8.0) | 251 (1.6) | 16,104 (64.2) |
| **Age (yrs)** | |  |  |  |  |
|  | Mean (SD) | 65.4 (9.1) | 66.1 (9.6) | 65.7 (9.8) | 65.4 (9.2) |
|  | 40-49 | 420 (94.4) | 19 (4.3) | 6 (1.3) | 445 (1.8) |
|  | 50-59 | 7050 (89.8) | 668 (8.5) | 129 (1.6) | 7847 (31.3) |
|  | 60-69 | 7905 (90.0) | 744 (8.5) | 135 (1.5) | 8784 (35.0) |
|  | 70-79 | 5709 (89.8) | 548 (8.6) | 99 (1.6) | 6356 (25.3) |
|  | 80- | 1427 (86.2) | 190 (11.5) | 38 (2.3) | 1655 (6.6) |
| **Region^b^** | |  |  |  |  |
|  | Qingdao (U) | 1598 (98.3) | 17 (1.0) | 10 (0.6) | 1625 (6.5) |
|  | Harbin (U) | 1658 (97.5) | 16 (0.9) | 27 (1.6) | 1701 (6.8) |
|  | Haikou (U) | 1274 (85.6) | 177 (11.9) | 38 (2.6) | 1489 (5.9) |
|  | Suzhou (U) | 2270 (82.3) | 460 (16.7) | 27 (1.0) | 2757 (11.0) |
|  | Liuzhou (U) | 2613 (94.9) | 63 (2.3) | 76 (2.8) | 2752 (11.0) |
|  | Sichuan (R) | 2849 (98.3) | 28 (1.0) | 20 (0.7) | 2897 (11.5) |
|  | Gansu (R) | 2572 (98.9) | 8 (0.3) | 20 (0.8) | 2600 (10.4) |
|  | Henan (R) | 3276 (98.9) | 14 (0.4) | 22 (0.7) | 3312 (13.2) |
|  | Zhejiang (R) | 1486 (50.9) | 1353 (46.4) | 80 (2.7) | 2919 (11.6) |
|  | Hunan (R) | 2915 (96.0) | 33 (1.1) | 87 (2.9) | 3035 (12.1) |
| **Marital status** | |  |  |  |  |
|  | Married | 18,443 (90.1) | 1724 (8.4) | 304 (1.5) | 20,471 (82.1) |
|  | Others | 4007 (90.0) | 343 (7.7) | 101 (2.3) | 4451 (17.9) |
| **Education** | |  |  |  |  |
|  | Primary school or below | 11,358 (87.4) | 1430 (11.0) | 209 (1.6) | 12,997 (51.8) |
|  | Middle school | 10,098 (92.0) | 704 (6.4) | 175 (1.6) | 10,977 (43.8) |
|  | College or above | 1055 (94.8) | 35 (3.1) | 23 (2.1) | 1113 (4.4) |
| **Household income level (yuan/year)** | |  |  |  |  |
|  | <20000 | 3428 (96.0) | 96 (2.7) | 48 (1.3) | 3572 (14.3) |
|  | 20000-75000 | 9897 (95.5) | 336 (3.2) | 132 (1.3) | 10,365 (41.6) |
|  | >75000 | 9125 (83.1) | 1635 (14.9) | 225 (2.0) | 10,985 (44.1) |
| **Occupation** | |  |  |  |  |
|  | Farmers | 2763 (92.8) | 191 (6.4) | 24 (0.8) | 2978 (11.9) |
|  | Factory worker | 1704 (81.0) | 365 (17.4) | 34 (1.6) | 2103 (8.4) |
|  | Office workers | 742 (91.2) | 58 (7.1) | 14 (1.7) | 814 (3.3) |
|  | Retired | 7546 (93.0) | 431 (5.3) | 135 (1.7) | 8112 (32.5) |
|  | House wife/husband | 6697 (90.5) | 568 (7.7) | 139 (1.9) | 7404 (29.7) |
|  | Service workers/Self-employed | 1867 (84.6) | 307 (13.9) | 34 (1.5) | 2208 (8.9) |
|  | Unemployed & others | 1131 (86.8) | 147 (11.3) | 25 (1.9) | 1303 (5.2) |
| **BMI (kg/m^2^)** | |  |  |  |  |
|  | <18.5 | 693 (86.0) | 103 (12.8) | 10 (1.2) | 806 (3.2) |
|  | 18.5-24 | 9562 (88.5) | 1055 (9.8) | 182 (1.7) | 10,799 (43.2) |
|  | 24-28 | 8848 (90.7) | 739 (7.6) | 165 (1.7) | 9752 (39.0) |
|  | >28 | 3398 (93.1) | 203 (5.6) | 48 (1.3) | 3649 (14.6) |

^a^ Participated: returned the accelerometer (i.e. “responded” in figure 1).

^b^ (U): urban region; (R): rural region.

##

## Supplementary Table 3. Wear-time compliance of the study population by demographic characteristics (N=21,894). The maximum possible wear time is 7.0 days.

| **Variables** | | **Wear-time [median (IQR) days]** | | |
| --- | --- | --- | --- | --- |
|  |  | **Men** | **Women** | **Overall** |
| **Total** | | 6.9 (6.0-7.0) | 6.9 (6.2-7.0) | 6.9 (6.1-7.0) |
| **Age (yrs)** | |  |  |  |
|  | 40-49 | 6.8 (5.8-7.0) | 6.8 (6.1-7.0) | 6.8 (6.0-7.0) |
|  | 50-59 | 6.8 (5.9-7.0) | 6.9 (6.2-7.0) | 6.9 (6.1-7.0) |
|  | 60-69 | 6.9 (6.1-7.0) | 6.9 (6.4-7.0) | 6.9 (6.2-7.0) |
|  | 70-79 | 6.9 (6.1-7.0) | 6.8 (6.3-7.0) | 6.8 (6.2-7.0) |
|  | 80- | 6.8 (6.0-7.0) | 6.8 (6.0-6.9) | 6.8 (6.0-6.9) |
| **Region** | |  |  |  |
|  | Qingdao | 6.9 (6.7-7.0) | 6.9 (6.7-7.0) | 6.9 (6.7-7.0) |
|  | Harbin | 6.8 (6.0-6.9) | 6.8 (6.1-6.9) | 6.8 (6.0-6.9) |
|  | Haikou | 6.1 (5.3-7.0) | 6.2 (5.9-7.0) | 6.2 (5.9-7.0) |
|  | Suzhou | 6.9 (6.2-7.0) | 6.9 (6.5-7.0) | 6.9 (6.4-7.0) |
|  | Liuzhou | 6.9 (6.3-7.0) | 6.9 (6.6-7.0) | 6.9 (6.6-7.0) |
|  | Sichuan | 6.9 (6.2-7.0) | 6.9 (6.4-7.0) | 6.9 (6.3-7.0) |
|  | Gansu | 6.8 (6.4-7.0) | 6.9 (6.6-7.0) | 6.9 (6.5-7.0) |
|  | Henan | 6.9 (6.3-7.0) | 6.8 (6.4-6.9) | 6.9 (6.3-6.9) |
|  | Zhejiang | 7.0 (6.3-7.0) | 6.9 (6.4-7.0) | 7.0 (6.3-7.0) |
|  | Hunan | 6.6 (5.7-6.9) | 6.7 (5.8-6.9) | 6.7 (5.8-6.9) |
| **Marital Status** | |  |  |  |
|  | Married | 6.9 (6.0-7.0) | 6.9 (6.2-7.0) | 6.9 (6.2-7.0) |
|  | Others | 6.8 (6.0-7.0) | 6.8 (6.2-7.0) | 6.8 (6.1-7.0) |
| **Education** | |  |  |  |
|  | Primary school or below | 6.9 (6.1-7.0) | 6.8 (6.2-7.0) | 6.9 (6.2-7.0) |
|  | Middle school | 6.9 (6.0-7.0) | 6.9 (6.3-7.0) | 6.9 (6.2-7.0) |
|  | College or above | 6.8 (5.9-7.0) | 6.8 (6.0-6.9) | 6.8 (6.0-7.0) |
| **Household Income Level (yuan/year)** | |  |  |  |
|  | <20000 | 6.8 (6.0-7.0) | 6.8 (6.2-7.0) | 6.8 (6.1-7.0) |
|  | 20000-75000 | 6.9 (6.1-7.0) | 6.9 (6.3-7.0) | 6.9 (6.2-7.0) |
|  | >75000 | 6.9 (6.0-7.0) | 6.9 (6.2-7.0) | 6.9 (6.1-7.0) |
| **Occupation** | |  |  |  |
|  | Farmers | 6.9 (6.2-7.0) | 6.9 (6.5-7.0) | 6.9 (6.4-7.0) |
|  | Factory worker | 6.9 (6.0-7.0) | 6.9 (6.1-7.0) | 6.9 (6.0-7.0) |
|  | Office workers | 6.8 (5.9-7.0) | 6.7 (6.0-6.9) | 6.7 (5.9-7.0) |
|  | Retired | 6.9 (6.1-7.0) | 6.9 (6.4-7.0) | 6.9 (6.3-7.0) |
|  | House wife/husband | 6.8 (5.9-7.0) | 6.8 (6.1-7.0) | 6.8 (6.1-7.0) |
|  | Service workers/Self-employed | 6.8 (6.0-7.0) | 6.9 (6.2-7.0) | 6.8 (6.1-7.0) |
|  | Unemployed & others | 6.8 (5.9-7.0) | 6.8 (6.0-6.9) | 6.8 (5.9-7.0) |
| **BMI (kg/m^2^)** | |  |  |  |
|  | <18.5 | 6.8 (5.9-7.0) | 6.8 (6.2-7.0) | 6.8 (6.0-7.0) |
|  | 18.5-24 | 6.8 (6.0-7.0) | 6.8 (6.1-7.0) | 6.8 (6.1-7.0) |
|  | 24-28 | 6.9 (6.1-7.0) | 6.9 (6.3-7.0) | 6.9 (6.2-7.0) |
|  | >28 | 6.9 (6.1-7.0) | 6.9 (6.4-7.0) | 6.9 (6.3-7.0) |

## Supplementary Table 4. Wear-time compliance of the study population by temporal characteristics (N=21,894).

| **Characteristics** | | **Wear-time [median (IQR) hours]** | | |
| --- | --- | --- | --- | --- |
|  |  | **Men** | **Women** | **Overall** |
| **Time of Day** ^a^ | |  |  |  |
|  | 0:00-5:59 | 6.0 (5.1-6.0) | 5.9 (5.1-6.0) | 6.0 (5.1-6.0) |
|  | 6:00-11:59 | 5.9 (5.4-6.0) | 5.9 (5.6-6.0) | 5.9 (5.5-6.0) |
|  | 12:00-17:59 | 5.9 (5.5-6.0) | 6.0 (5.6-6.0) | 6.0 (5.6-6.0) |
|  | 18:00-23:59 | 6.0 (5.2-6.0) | 6.0 (5.5-6.0) | 6.0 (5.4-6.0) |
| **Day of Week** ^b^ | |  |  |  |
|  | Mon | 24.0 (22.8-24.0) | 24.0 (22.9-24.0) | 24.0 (22.9-24.0) |
|  | Tue | 24.0 (22.6-24.0) | 24.0 (22.9-24.0) | 24.0 (22.8-24.0) |
|  | Wed | 24.0 (22.8-24.0) | 24.0 (22.9-24.0) | 24.0 (22.8-24.0) |
|  | Thu | 24.0 (22.8-24.0) | 24.0 (22.9-24.0) | 24.0 (22.9-24.0) |
|  | Fri | 24.0 (22.9-24.0) | 24.0 (23.0-24.0) | 24.0 (23.0-24.0) |
|  | Sat | 24.0 (24.0-24.0) | 24.0 (23.5-24.0) | 24.0 (23.6-24.0) |
|  | Sun | 24.0 (23.3-24.0) | 24.0 (23.0-24.0) | 24.0 (23.1-24.0) |

^a^ Sum wear-time hours for time quadrant of a day displayed (max=6.0 hours).

^b^ Median wear-time hours for day displayed (max=24.0 hours).

## Supplementary Table 5. Median (IQR) levels of movement behaviours (N=20,370)

| **Variables** | | **Average acceleration (mg/d)** | **MVPA (min/d)** | **LIPA (h/d)** | **Sedentary (h/d)** | **Sleep (h/d)** |
| --- | --- | --- | --- | --- | --- | --- |
| **Total** | | 29.7 (22.6-37.9) | 88.4 (48.2-142.4) | 5.7 (4.3-7.1) | 8.7 (6.7-10.7) | 7.7 (6.9-8.6) |
| **Age (yrs)** | |  |  |  |  |  |
|  | 40-49 | 35.5 (28.7-43.7) | 129.0 (85.6-184.3) | 5.7 (4.2-7.1) | 8.3 (6.4-10.2) | 7.5 (6.8-8.3) |
|  | 50-59 | 34.3 (27.5-42.7) | 119.3 (77.2-178.7) | 6.0 (4.8-7.4) | 7.8 (6.0-9.7) | 7.7 (6.9-8.4) |
|  | 60-69 | 30.4 (23.9-38.1) | 92.8 (56.0-142.4) | 5.9 (4.5-7.3) | 8.5 (6.6-10.4) | 7.7 (6.9-8.5) |
|  | 70-79 | 24.9 (18.8-32.1) | 59.1 (29.6-104.0) | 5.3 (3.9-6.8) | 9.5 (7.5-11.5) | 7.8 (6.9-8.8) |
|  | 80- | 18.1 (13.5-23.8) | 23.4 (8.6-52.3) | 4.3 (2.6-5.9) | 10.9 (9.1-13.0) | 8.1 (6.9-9.2) |
| **Sex** | |  |  |  |  |  |
|  | Men | 26.7 (20.2-34.7) | 76.1 (40.6-125.0) | 5.0 (3.6-6.4) | 9.6 (7.6-11.6) | 7.8 (6.9-8.7) |
|  | Women | 31.2 (24.1-39.3) | 95.3 (53.2-151.4) | 6.1 (4.8-7.4) | 8.2 (6.3-10.1) | 7.7 (6.9-8.5) |
| **Marital Status** | |  |  |  |  |  |
|  | Married | 30.4 (23.3-38.6) | 93.5 (52.8-147.7) | 5.8 (4.4-7.2) | 8.5 (6.6-10.5) | 7.7 (6.9-8.6) |
|  | Others | 26.3 (19.3-34.4) | 65.0 (30.1-117.3) | 5.4 (4.0-6.9) | 9.3 (7.3-11.3) | 7.7 (6.8-8.7) |
| **Education** | |  |  |  |  |  |
|  | Primary school or below | 29.4 (21.8-38.2) | 83.3 (40.7-142.0) | 5.9 (4.5-7.4) | 8.3 (6.3-10.4) | 7.9 (7.0-8.8) |
|  | Middle school | 30.1 (23.4-38.1) | 94.5 (54.9-145.8) | 5.6 (4.2-6.9) | 8.8 (7.0-10.8) | 7.6 (6.8-8.4) |
|  | College or above | 28.0 (22.0-33.8) | 85.8 (54.5-125.1) | 4.7 (3.6-5.8) | 10.4 (9.0-11.9) | 7.2 (6.5-8.0) |
| **Household Income Level (yuan/year)** | |  |  |  |  |  |
|  | <20000 | 28.3 (20.5-37.0) | 78.4 (37.1-134.0) | 5.7 (4.2-7.2) | 8.5 (6.5-10.6) | 8.0 (7.1-9.0) |
|  | 20000-75000 | 29.6 (22.5-38.0) | 88.0 (47.6-143.7) | 5.7 (4.3-7.1) | 8.6 (6.7-10.7) | 7.7 (6.9-8.7) |
|  | >75000 | 30.2 (23.3-38.1) | 92.4 (52.7-143.4) | 5.7 (4.3-7.2) | 8.8 (6.8-10.8) | 7.6 (6.8-8.4) |
| **Occupation** | |  |  |  |  |  |
|  | Farmers | 35.5 (27.9-44.7) | 124.4 (78.4-190.8) | 6.3 (5.1-7.5) | 7.1 (5.5-8.9) | 7.9 (7.1-8.8) |
|  | Factory worker | 36.2 (28.6-45.5) | 130.6 (81.0-202.0) | 6.4 (5.1-7.8) | 7.0 (5.1-9.1) | 7.7 (7.0-8.5) |
|  | Office workers | 29.1 (23.8-34.9) | 94.3 (61.4-130.0) | 4.7 (3.6-6.0) | 10.0 (8.5-11.8) | 7.4 (6.6-8.2) |
|  | Retired | 26.7 (20.5-33.8) | 72.8 (38.7-117.4) | 5.1 (3.8-6.5) | 9.8 (8.1-11.7) | 7.4 (6.6-8.3) |
|  | House wife/husband | 29.2 (22.2-37.0) | 82.5 (42.6-135.2) | 6.0 (4.7-7.3) | 8.2 (6.6-10.1) | 7.9 (7.1-8.8) |
|  | Service workers/Self-employed | 34.1 (27.1-42.0) | 113.2 (72.2-172.1) | 6.5 (5.1-7.8) | 7.5 (5.7-9.4) | 7.7 (6.9-8.4) |
|  | Unemployed & others | 22.8 (15.5-31.6) | 50.3 (17.7-104.9) | 4.4 (2.8-6.1) | 10.2 (8.1-12.2) | 8.1 (7.1-9.1) |
| **BMI (kg/m^2^)** | |  |  |  |  |  |
|  | <18.5 | 28.8 (20.2-39.2) | 79.6 (36.0-150.2) | 5.7 (4.0-7.2) | 8.5 (6.6-10.7) | 7.9 (7.0-8.9) |
|  | 18.5-24 | 31.0 (23.3-39.4) | 95.9 (52.2-154.4) | 5.9 (4.4-7.3) | 8.2 (6.3-10.3) | 7.8 (7.0-8.7) |
|  | 24-28 | 29.6 (22.9-37.5) | 88.8 (49.8-139.4) | 5.7 (4.4-7.1) | 8.7 (6.9-10.7) | 7.7 (6.8-8.6) |
|  | >28 | 26.7 (20.7-34.4) | 71.6 (37.9-118.8) | 5.3 (3.9-6.8) | 9.6 (7.6-11.6) | 7.5 (6.6-8.4) |
| **Urban** | |  |  |  |  |  |
|  | Qingdao | 26.9 (20.9-33.8) | 77.2 (42.7-121.0) | 4.8 (3.6-6.0) | 10.2 (8.6-12.0) | 7.4 (6.6-8.1) |
|  | Harbin | 25.2 (19.6-31.8) | 69.0 (38.5-108.9) | 4.6 (3.4-5.9) | 10.8 (9.2-12.5) | 7.1 (6.3-7.9) |
|  | Haikou | 34.6 (26.7-44.6) | 121.5 (72.5-181.8) | 5.6 (4.3-7.0) | 8.4 (6.6-10.4) | 7.5 (6.6-8.3) |
|  | Suzhou | 29.2 (22.6-36.7) | 78.3 (43.7-128.2) | 6.3 (4.7-7.8) | 8.1 (6.3-10.2) | 7.8 (7.0-8.6) |
|  | Liuzhou | 27.9 (22.1-34.7) | 81.3 (49.0-124.1) | 5.4 (4.1-6.6) | 9.6 (8.1-11.2) | 7.3 (6.5-8.1) |
|  | Total | 28.3 (21.9-35.6) | 81.9 (46.1-129.0) | 5.3 (4.0-6.8) | 9.5 (7.6-11.4) | 7.4 (6.6-8.2) |
| **Rural** |  |  |  |  |  |  |
|  | Sichuan | 33.6 (25.7-42.8) | 111.4 (62.4-181.5) | 6.1 (5.0-7.3) | 7.1 (5.4-9.0) | 8.3 (7.5-9.2) |
|  | Gansu | 31.5 (23.8-40.1) | 98.2 (55.5-155.1) | 5.9 (4.4-7.4) | 8.6 (6.8-10.6) | 7.4 (6.6-8.3) |
|  | Henan | 27.1 (21.0-34.4) | 74.1 (39.7-124.1) | 5.7 (4.4-6.9) | 8.4 (6.7-10.2) | 8.3 (7.4-9.1) |
|  | Zhejiang | 35.4 (26.9-44.8) | 120.2 (71.9-189.3) | 6.6 (5.3-8.0) | 7.0 (5.0-9.2) | 7.7 (7.0-8.6) |
|  | Hunan | 30.1 (21.9-38.4) | 84.6 (41.2-141.9) | 5.9 (4.3-7.4) | 8.5 (6.6-10.6) | 7.8 (7.0-8.6) |
|  | Total | 30.9 (23.2-39.3) | 94.3 (49.6-153.6) | 6.0 (4.6-7.3) | 8.0 (6.2-10.1) | 7.9 (7.1-8.8) |
| **Time of Day** | |  |  |  |  |  |
|  | 0:00-5:59 | 1.3 (1.0-2.1) | 0.8 (0.1-3.3) | 0.1 (0.0-0.3) | 0.6 (0.3-1.0) | 5.2 (4.6-5.6) |
|  | 6:00-11:59 | 11.4 (8.4-15.0) | 37.5 (19.7-62.1) | 2.4 (1.8-3.0) | 2.1 (1.4-2.9) | 0.5 (0.2-0.9) |
|  | 12:00-17:59 | 9.5 (7.0-12.7) | 26.1 (12.9-46.3) | 2.0 (1.3-2.6) | 3.0 (2.2-3.8) | 0.3 (0.0-0.7) |
|  | 18:00-23:59 | 5.9 (3.9-8.6) | 14.7 (5.8-29.8) | 1.0 (0.6-1.5) | 2.8 (2.1-3.4) | 1.7 (1.0-2.4) |
| **Day of Week** | |  |  |  |  |  |
|  | Mon | 29.1 (21.7-38.3) | 84.5 (42.0-145.0) | 5.7 (4.0-7.3) | 8.6 (6.3-11.0) | 7.7 (6.5-8.9) |
|  | Tue | 28.8 (21.4-38.1) | 82.5 (41.0-142.0) | 5.6 (4.0-7.3) | 8.6 (6.4-11.0) | 7.7 (6.6-8.9) |
|  | Wed | 28.9 (21.4-38.2) | 83.5 (41.0-143.0) | 5.6 (4.0-7.3) | 8.6 (6.3-10.9) | 7.7 (6.6-9.0) |
|  | Thu | 29.0 (21.5-38.1) | 83.0 (41.0-143.0) | 5.6 (4.0-7.3) | 8.6 (6.4-11.0) | 7.7 (6.6-9.0) |
|  | Fri | 29.1 (21.6-38.5) | 83.5 (41.5-144.0) | 5.7 (4.0-7.4) | 8.7 (6.3-11.0) | 7.6 (6.5-8.9) |
|  | Sat | 28.9 (21.4-38.3) | 81.0 (40.0-143.5) | 5.7 (4.0-7.4) | 8.7 (6.4-11.1) | 7.6 (6.4-8.8) |
|  | Sun | 28.9 (21.2-38.3) | 81.5 (39.0-142.0) | 5.6 (4.0-7.4) | 8.7 (6.3-11.1) | 7.6 (6.5-8.8) |

MVPA: moderate-to-vigorous physical activity behaviours; LIPA: light-intensity physical activity behaviours

## Supplementary Table 6. Mean (SE) levels of movement behaviours by regions ^a^ (N=20,370)

|  | **Region** | **Average acceleration (mg/d)** | **MVPA (min/d) ^b^** | **LIPA (h/d)** | **Sedentary (h/d)** | **Sleep (h/d)** |
| --- | --- | --- | --- | --- | --- | --- |
| Urban | Qingdao | 29.5 (0.28) | 77.7 (0.02) | 4.8 (0.05) | 10.2 (0.07) | 7.4 (0.04) |
|  | Harbin | 26.1 (0.27) | 57.0 (0.02) | 4.7 (0.05) | 10.9 (0.06) | 7.1 (0.04) |
|  | Haikou | 35.6 (0.31) | 100.6 (0.02) | 5.6 (0.06) | 8.8 (0.07) | 7.5 (0.04) |
|  | Suzhou | 30.1 (0.23) | 65.7 (0.02) | 6.3 (0.04) | 8.4 (0.06) | 7.8 (0.03) |
|  | Liuzhou | 30.1 (0.23) | 77.9 (0.02) | 5.5 (0.04) | 9.6 (0.06) | 7.3 (0.03) |
|  | Total | 30.0 (0.12) | 72.8 (0.01) | 5.4 (0.02) | 9.5 (0.03) | 7.4 (0.02) |
| Rural | Sichuan | 35.0 (0.21) | 95.2 (0.02) | 6.1 (0.04) | 7.4 (0.05) | 8.4 (0.03) |
|  | Gansu | 31.3 (0.22) | 72.4 (0.02) | 5.8 (0.04) | 9.0 (0.05) | 7.5 (0.03) |
|  | Henan | 27.8 (0.19) | 58.7 (0.01) | 5.6 (0.04) | 8.6 (0.05) | 8.3 (0.03) |
|  | Zhejiang | 37.7 (0.29) | 113.1 (0.02) | 6.8 (0.05) | 7.1 (0.07) | 7.8 (0.04) |
|  | Hunan | 31.0 (0.21) | 68.7 (0.02) | 5.9 (0.04) | 8.6 (0.05) | 7.8 (0.03) |
|  | Total | 31.9 (0.10) | 75.7 (0.01) | 5.9 (0.02) | 8.3 (0.02) | 8.0 (0.01) |

MVPA: moderate-to-vigorous physical activity behaviours; LIPA: light-intensity physical activity behaviours

^a^ Values were adjusted for age and sex.

^b^ Level of MVPA was reported based on logarithmic transformation.

## Supplementary Table 7. Mean (SE) levels of movement behaviours by temporal characteristics (N=20,370)

| **Variables** | | **Average acceleration (mg/d)** | **MVPA (min/d)** | **LIPA(h/d)** | **Sedentary (h/d)** | **Sleep (h/d)** |
| --- | --- | --- | --- | --- | --- | --- |
| **Time of Day** | |  |  |  |  |  |
|  | 0:00-5:59 | 1.8 (0.01) | 3.5 (0.06) | 0.2 (0.00) | 0.8 (0.00) | 5.0 (0.01) |
|  | 6:00-11:59 | 12.2 (0.04) | 45.3 (0.25) | 2.4 (0.01) | 2.2 (0.01) | 0.6 (0.00) |
|  | 12:00-17:59 | 10.3 (0.03) | 34.7 (0.22) | 2.0 (0.01) | 3.0 (0.01) | 0.4 (0.00) |
|  | 18:00-23:59 | 6.8 (0.03) | 20.9 (0.14) | 1.1 (0.00) | 2.8 (0.01) | 1.7 (0.01) |
| **Day of Week** | |  |  |  |  |  |
|  | Mon | 31.3 (0.10) | 105.8 (0.62) | 5.7 (0.02) | 8.7 (0.02) | 7.8 (0.01) |
|  | Tue | 31.0 (0.10) | 104.2 (0.62) | 5.7 (0.02) | 8.7 (0.02) | 7.8 (0.01) |
|  | Wed | 31.0 (0.10) | 104.1 (0.61) | 5.7 (0.02) | 8.7 (0.02) | 7.8 (0.01) |
|  | Thu | 31.0 (0.10) | 104.1 (0.61) | 5.7 (0.02) | 8.8 (0.02) | 7.8 (0.01) |
|  | Fri | 31.2 (0.10) | 105.0 (0.62) | 5.8 (0.02) | 8.8 (0.02) | 7.7 (0.01) |
|  | Sat | 31.1 (0.10) | 103.5 (0.62) | 5.8 (0.02) | 8.9 (0.02) | 7.6 (0.01) |
|  | Sun | 31.0 (0.10) | 103.8 (0.63) | 5.7 (0.02) | 8.8 (0.02) | 7.7 (0.01) |

MVPA: moderate-to-vigorous physical activity behaviours; LIPA: light-intensity physical activity behaviours

## Supplementary Table 8. Characteristics of the UK Biobank accelerometer dataset [N(%)].

|  |  | **Men** | **Women** | **Overall** |
| --- | --- | --- | --- | --- |
| **N (%)** | | 42,080 (43.7) | 54,233 (56.4) | 96,313 |
| **Age (yrs)** | |  |  |  |
|  | Mean (SD) | 63.1 (7.9) | 61.9 (7.8) | 62.5 (7.8) |
|  | 40-49 | 3,237 (7.7) | 4,597 (8.5) | 7,834 (8.1) |
|  | 50-59 | 10,741 (25.5) | 16,645 (30.7) | 27,386 (28.4) |
|  | 60-69 | 18,829 (44.7) | 24,051 (44.3) | 42,880 (44.5) |
|  | 70-79 | 9,273 (22.0) | 8,940 (16.5) | 18,213 (18.9) |

## Supplementary Table 9. Median (IQR) levels of movement behaviours in the UK Biobank accelerometer dataset (N= 96,313).

| **Variables** | | **Average acceleration (mg/d)** | **MVPA (min/d)** | **LIPA (h/d)** | **Sedentary (h/d)** | **Sleep (h/d)** |
| --- | --- | --- | --- | --- | --- | --- |
| **Total** | | 27.1 (22.5-32.5) | 88.1 (59.8-123.0) | 4.3 (3.4-5.3) | 10.2 (9.0-11.5) | 7.8 (7.2-8.4) |
| **Age (yrs)** | |  |  |  |  |  |
|  | 40-49 | 30.5 (25.5-36.6) | 105.9 (76.8-141.8) | 4.1 (3.2-5.1) | 10.2 (8.8-11.5) | 7.8 (7.2-8.4) |
|  | 50-59 | 29.1 (24.3-34.7) | 98.7 (70.4-134.4) | 4.1 (3.2-5.2) | 10.2 (8.9-11.5) | 7.8 (7.2-8.3) |
|  | 60-69 | 26.6 (22.2-31.8) | 86.1 (58.4-120.3) | 4.4 (3.5-5.4) | 10.2 (8.9-11.4) | 7.8 (7.2-8.4) |
|  | 70-79 | 24.2 (20.1-28.7) | 69.4 (44.4-99.3) | 4.4 (3.5-5.4) | 10.5 (9.3-11.8) | 7.7 (7.1-8.4) |
| **Sex** | |  |  |  |  |  |
|  | Men | 26.5 (21.8-32.0) | 84.6 (57.4-118.6) | 4.0 (3.1-5.0) | 10.8 (9.5-12.1) | 7.6 (7.0-8.2) |
|  | Women | 27.6 (23.1-32.9) | 90.9 (61.8-126.4) | 4.5 (3.7-5.5) | 9.8 (8.6-11.1) | 7.9 (7.3-8.5) |

## Supplementary Table 10. Confusion matrices of the machine learning classifier in free-living environments: the CAPTURE-24CN and CAPTURE-24 studies. Minutes shown in brackets.

| Test on Chinese CAPTURE-24CN  Cohen’s kappa: .674 (.614, .743)  Accuracy: .814 (.783, .851)  N = 44 | | | |
| --- | --- | --- | --- |
| **Observed\Model** | **sleep** | **sedentary** | **other** |
| **sleep** | 91% (14,669) | 8% (1,168) | 2% (292) |
| **sedentary** | 2% (265) | 73% (10,261) | 24% (3,533) |
| **other** | 1% (104) | 20% (2,871) | 80% (12,049) |

| Test on UK CAPTURE-24 (Leave-one-out cross-validation)  Cohen’s kappa: .840 (.823, .855)  Accuracy: .900 (.891, .913)  N = 151 | | | |
| --- | --- | --- | --- |
| **Observed\Model** | **sleep** | **sedentary** | **other** |
| **sleep** | 97% (55,201) | 2% (1,283) | <1% (194) |
| **sedentary** | 1% (373) | 85% (52,079) | 14% (8,509) |
| **other** | 0% (0) | 12% (4365) | 88% (31,616) |

## Supplementary Figure 1. Flowchart of the process of the accelerometer data collection.


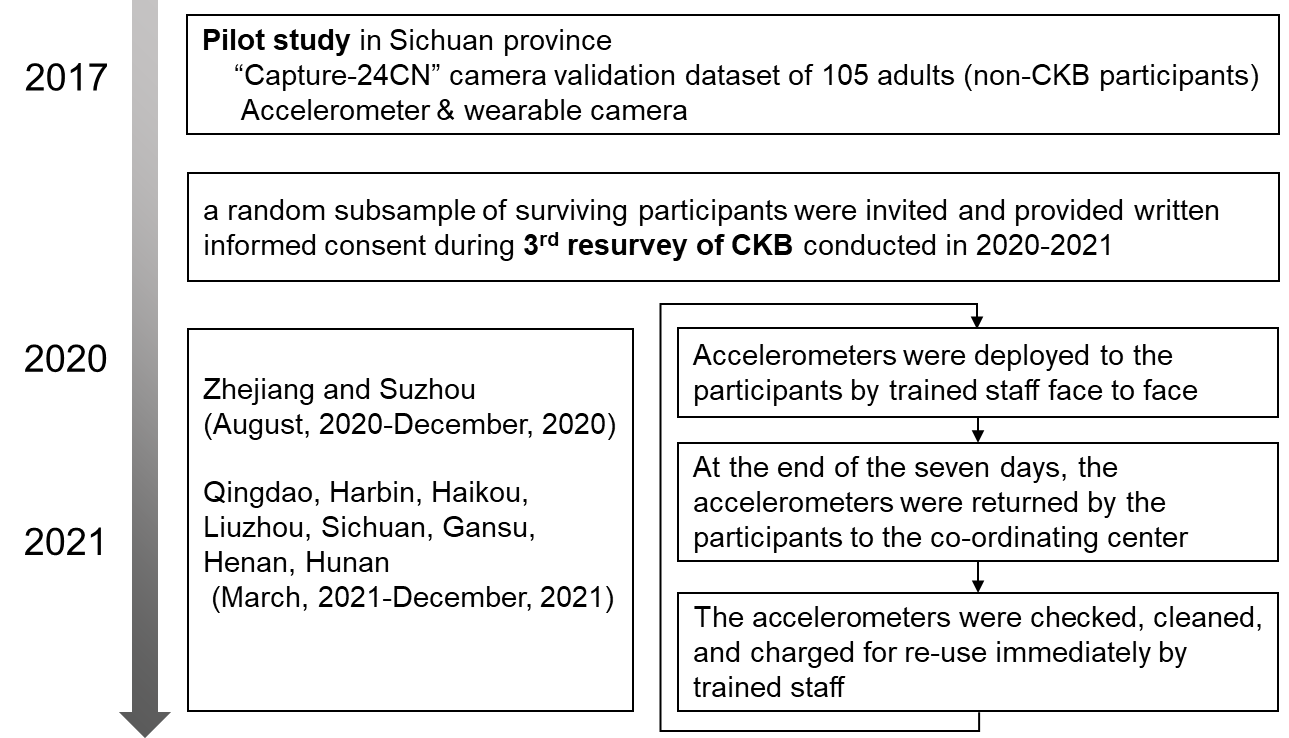


## Supplementary Figure 2. Start/end date of fieldwork across 10 study regions*.

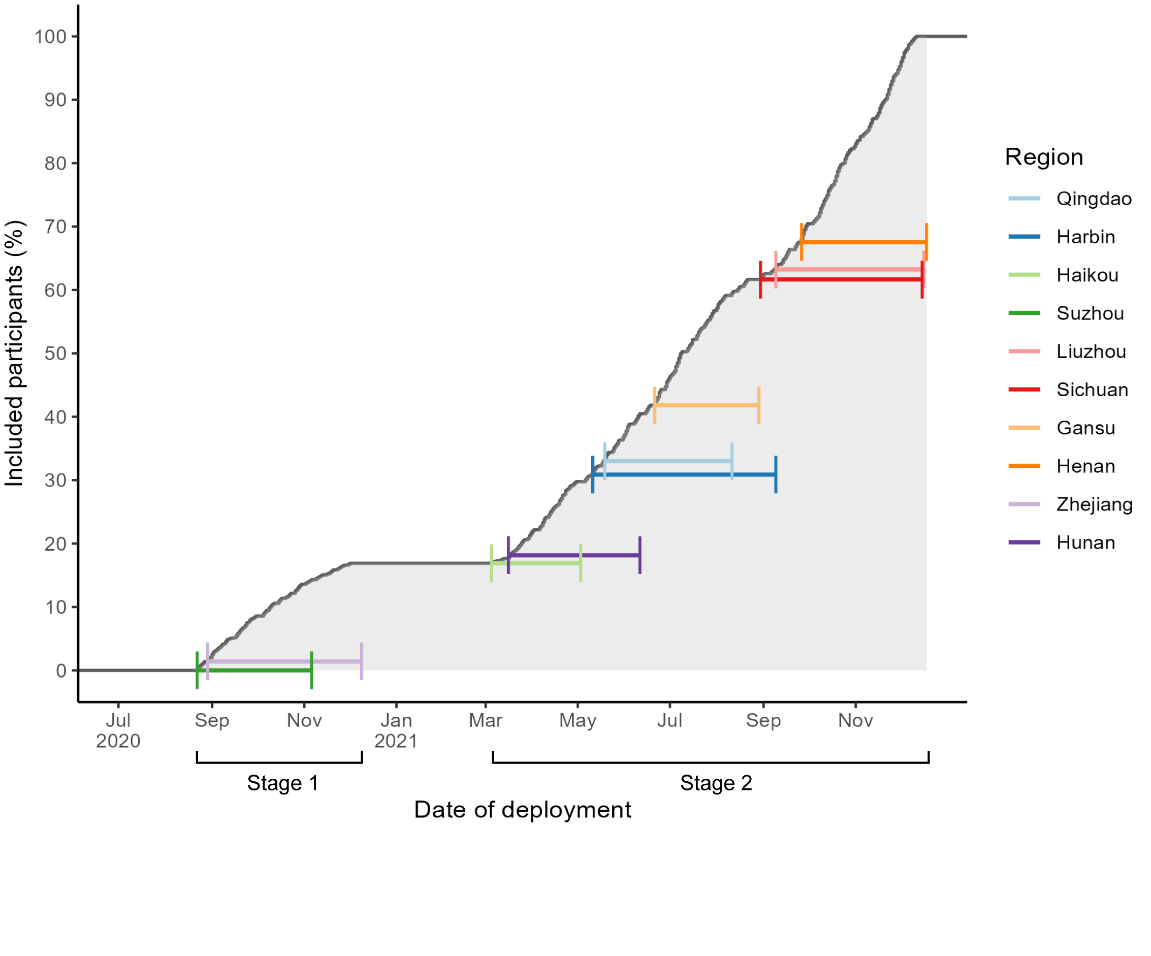


*The black line against Y-axis represents the cumulative proportion of included participants and the coloured horizontal bars indicate the time period of the accelerometer data collection.

## Supplementary Figure 3. 24-h profile of four movement behaviours by age group^*^.


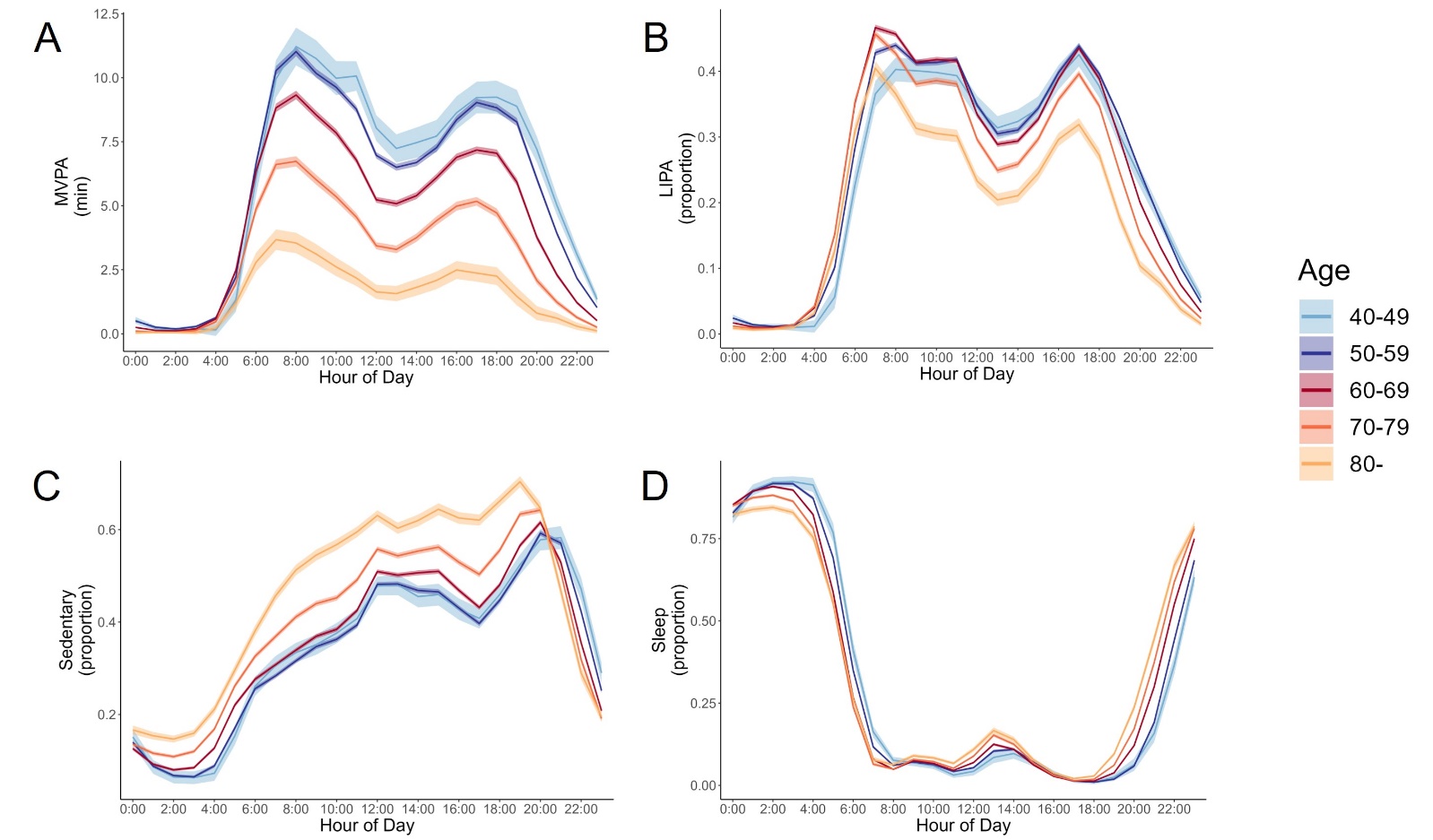


(A) moderate-to-vigorous physical activity time (MVPA, min); (B) light-intensity physical activity time (LIPA, proportion); (C) sedentary time (proportion); (D) sleep time (proportion).

^*^ Values were adjusted for sex and region. The ribbon represents the 95% confidence interval.

## Supplementary Figure 4. 24-h profile of four movement behaviours by sex.


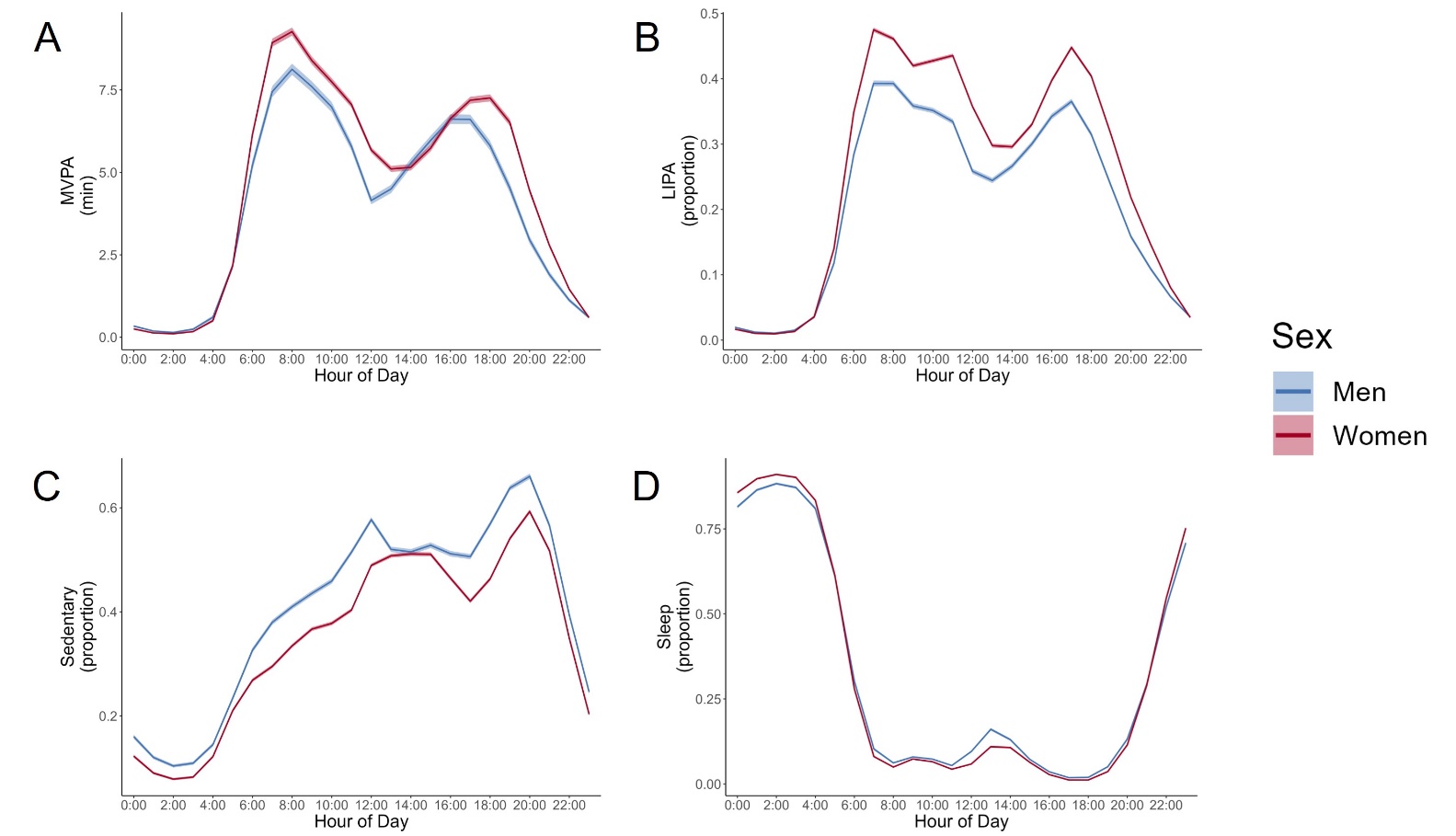


(A) moderate-to-vigorous physical activity time (MVPA, min); (B) light-intensity physical activity time (LIPA, proportion); (C) sedentary time (proportion); (D) sleep time (proportion).

^*^ Values were adjusted for age and region. The ribbon represents the 95% confidence interval.

## Supplementary Figure 5. 24-h profile of different movement behaviours by region^*^.


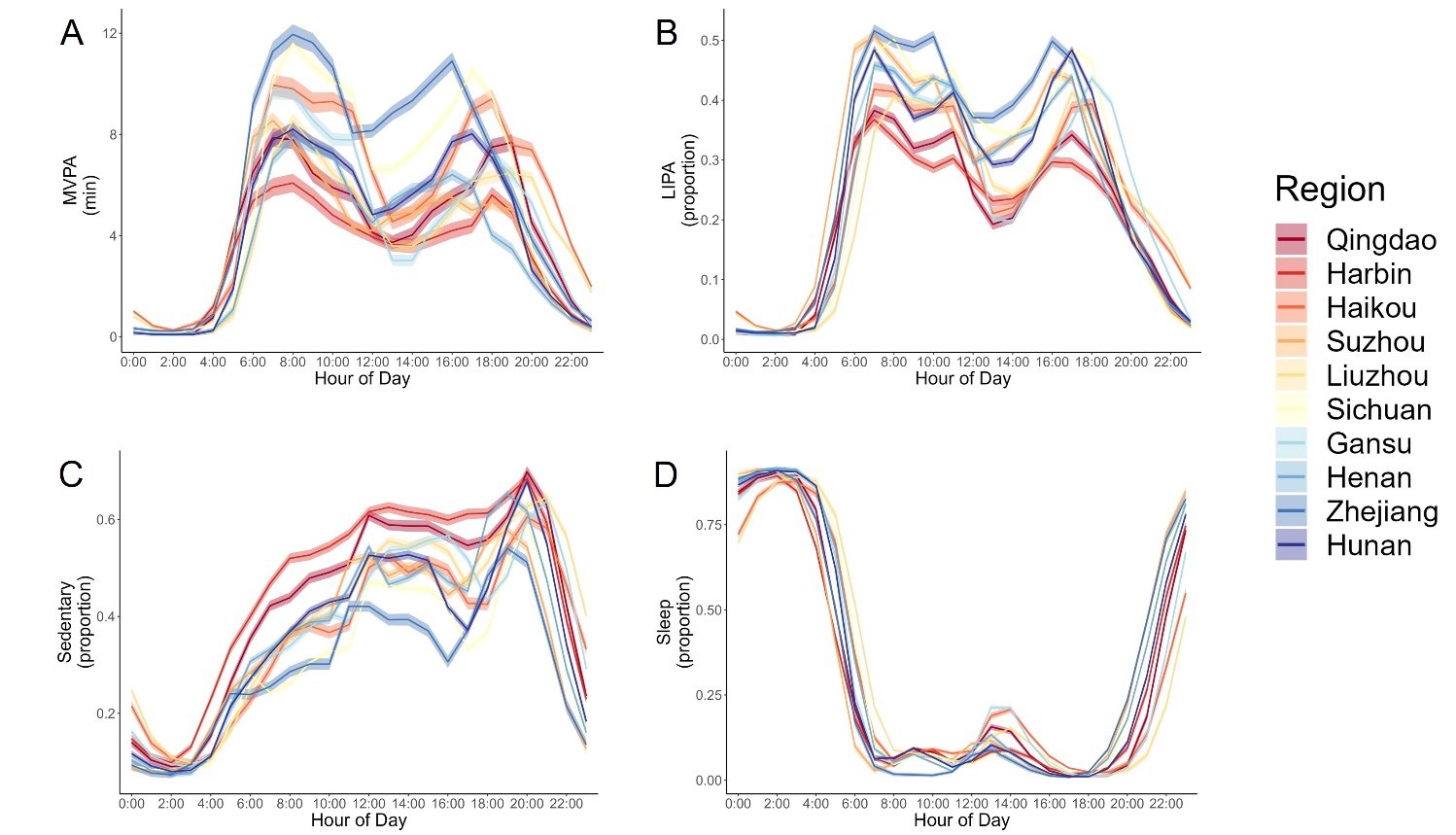


(A) moderate-to-vigorous physical activity time (MVPA, min); (B) light-intensity physical activity time (LIPA, proportion); (C) sedentary time (proportion); (D) sleep time (proportion).

^*^ Values were adjusted for age and sex. The ribbon represents the 95% confidence interval.

## Supplementary Figure 6. The process of model development, validation and deployment.

**
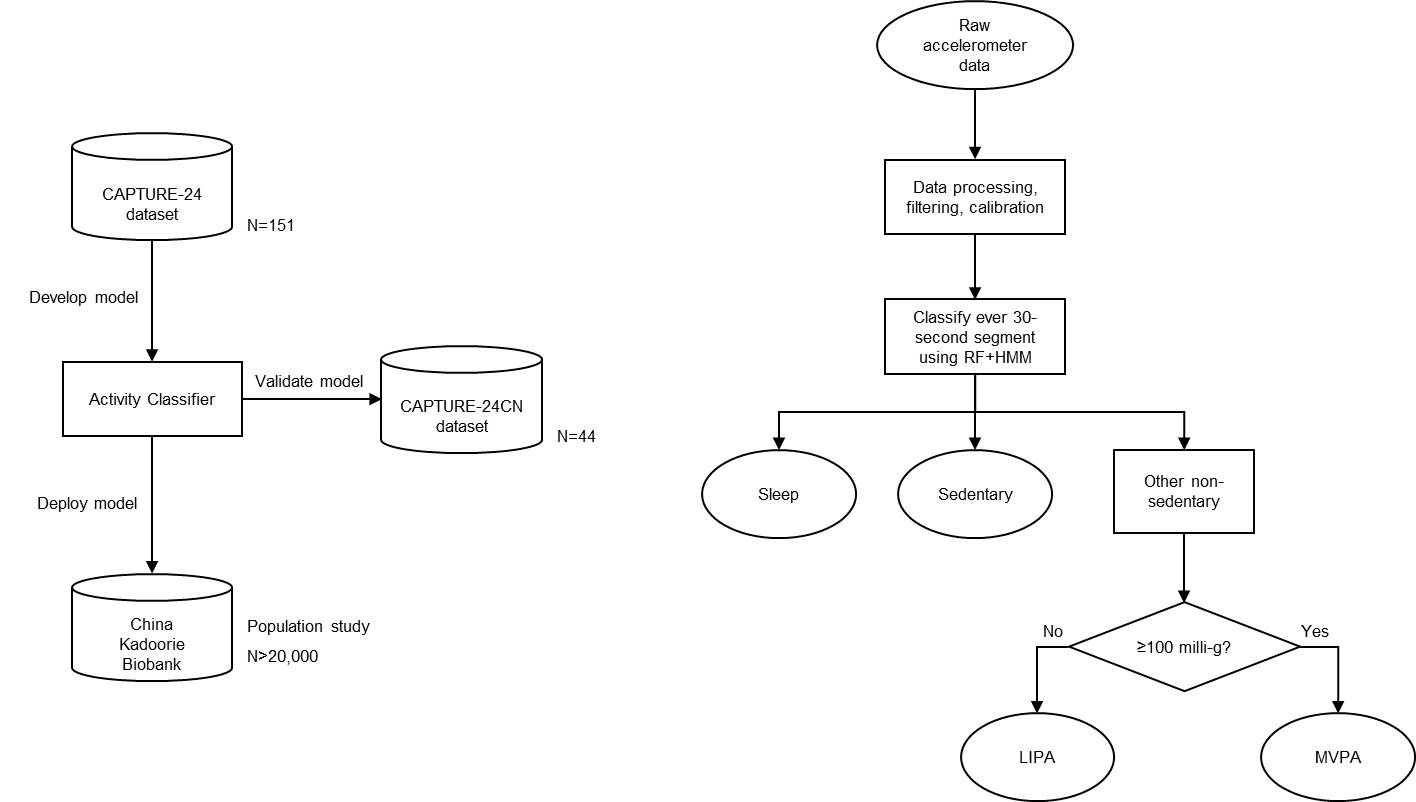
**

RF: Balanced Random Forest; HMM: Hidden Markov model; MVPA: moderate-to-vigorous physical activity time; LIPA: light-intensity physical activity time.
